# Lytic Reactivation of the Kaposi’s Sarcoma-Associated Herpesvirus (KSHV) Is Accompanied by Major Nucleolar Alterations

**DOI:** 10.3390/v14081720

**Published:** 2022-08-04

**Authors:** Nofar Atari, K. Shanmugha Rajan, Vaibhav Chikne, Smadar Cohen-Chalamish, Tirza Doniger, Odelia Orbaum, Avi Jacob, Inna Kalt, Shulamit Michaeli, Ronit Sarid

**Affiliations:** The Mina and Everard Goodman Faculty of Life Sciences, Advanced Materials and Nanotechnology Institute, Bar-Ilan University, Ramat-Gan 5290002, Israel

**Keywords:** Kaposi’s sarcoma-associated herpesvirus, KSHV, nucleolus, upstream binding factor, UBF, Fibrillarin, FBL, nucleophosmin (NPM1/B23), ribosomal RNA (rRNA), pseudouridylation, 2′-*O*-methylation

## Abstract

The nucleolus is a subnuclear compartment whose primary function is the biogenesis of ribosomal subunits. Certain viral infections affect the morphology and composition of the nucleolar compartment and influence ribosomal RNA (rRNA) transcription and maturation. However, no description of nucleolar morphology and function during infection with Kaposi’s sarcoma-associated herpesvirus (KSHV) is available to date. Using immunofluorescence microscopy, we documented extensive destruction of the nuclear and nucleolar architecture during the lytic reactivation of KSHV. This was manifested by the redistribution of key nucleolar proteins, including the rRNA transcription factor UBF. Distinct delocalization patterns were evident; certain nucleolar proteins remained together whereas others dissociated, implying that nucleolar proteins undergo nonrandom programmed dispersion. Significantly, the redistribution of UBF was dependent on viral DNA replication or late viral gene expression. No significant changes in pre-rRNA levels and no accumulation of pre-rRNA intermediates were found by RT-qPCR and Northern blot analysis. Furthermore, fluorescent in situ hybridization (FISH), combined with immunofluorescence, revealed an overlap between Fibrillarin and internal transcribed spacer 1 (ITS1), which represents the primary product of the pre-rRNA, suggesting that the processing of rRNA proceeds during lytic reactivation. Finally, small changes in the levels of pseudouridylation (Ψ) and 2′-*O*-methylation (Nm) were documented across the rRNA; however, none were localized to the functional domain. Taken together, our results suggest that despite dramatic changes in the nucleolar organization, rRNA transcription and processing persist during lytic reactivation of KSHV. Whether the observed nucleolar alterations favor productive infection or signify cellular anti-viral responses remains to be determined.

## 1. Introduction

KSHV, also known as human herpesvirus 8 (HHV-8), is a cancer-related gamma-2 herpesvirus which is etiologically implicated in all types of Kaposi’s sarcoma (KS). In addition, KSHV is the causative agent of other disorders, including primary effusion lymphoma (PEL), multicentric Castleman’s disease, and KSHV-inflammatory cytokine syndrome [1,2,3,4,5,6]. As with all herpesviruses, KSHV undergoes either lytic (productive) or latent infection. During latent infection, the virus becomes largely transcriptionally quiescent and no virions are produced. In contrast, the productive course involves the extensive transcription, replication, assembly and packaging of viral DNA, and ends with the release of new virions. The productive infection takes place in the host cell nucleus and is accompanied by drastic reorganization of the nuclear architecture involving conformational changes of the nuclear lamina and the positioning of condensed chromatin at the nuclear periphery. At the same time, globular viral replication compartments (RCs), devoid of chromatin, are formed and then merge into a kidney-shaped zone characterized by high levels of viral DNA synthesis and high concentrations of viral and host proteins. Viral transcriptional factories, which recruit a significant fraction of RNA polymerase II, also accumulate in the nucleus, mostly in foci in and around RCs [7,8,9]. These events have been comprehensively studied for certain herpesviruses [10,11,12,13], yet the characteristics of nuclear alterations, particularly those in the nucleolus, during KSHV infection have only begun to be elucidated.

The nucleolus is a non-membrane bound subnuclear structure whose primary function is the biogenesis of ribosomal subunits, accomplished by the transcription of pre-ribosomal RNA (pre-rRNA), rRNA processing and modifications and assembly with ribosomal proteins. These functions are reflected by three distinct nucleolar substructures known as the fibrillar center (FC), the dense fibrillar component (DFC), and the granular component (GS). The nucleolus is surrounded by heterochromatin, forming perinucleolar chromatin [14,15,16,17,18,19]. The FC is rich in upstream binding factor (UBF), which plays a role in the recruitment of RNA polymerase I to rDNA promoters, and in chromatin structure modulation. Transcription of pre-rRNA takes place either in the FC or at the boundary between the FC and the DFC. The post-transcriptional processing and modifications of pre-rRNA occur in the DFC region, which contains processing factors such as the small nucleolar-associated enzyme 2′-*O*-methyltransferase Fibrillarin, and the pseudouridine synthase Dyskerin (DKC1). Late rRNA processing, along with assembly and transport of ribosomal subunits, occurs in the GC region, which comprises the remainder of the nucleolus and is enriched in processing and assembly factors such as Nucleophosmin 1 (NPM1/B23) and Nucleolin, as well as ribosomal proteins. The nucleolus is also involved in other cellular processes that may not be directly associated with the biogenesis of ribosomal subunits, including cell cycle control, stress responses, senescence, telomerase activity, protein degradation and sequestration, chromosomal domain organization, and innate immune responses [20,21]. Hence, the nucleolus has a dynamic composition of proteins and RNA molecules with diverse functions.

Viral infections may cause nucleolar alterations, manifested by the redistribution of host nucleolar molecules to different cellular sites, the occurrence of distinct RNA or protein modifications, and the targeting of cellular proteins to the nucleoli. In addition, viral products may traffic to and from the nucleolus, and thereby alter the cell cycle, mRNA transport and innate host cell immunity. A large proportion of these activities are not restricted to any particular type of virus [19,22,23,24,25,26,27]. For example, the replication and transcription of the Borna disease virus take place in the nucleolus [28], assembly of AAV occurs in the nucleolus [29], REV protein of HIV is localized to the nucleolus [30,31], and modifications of the basal rRNA transcription factors, Selectivity Factor-1 (SL-1) and UBF, inhibit the activity of RNA polymerase I during poliovirus infection [32,33]. In addition, the non-structural protein NSs, encoded by the Schmallenberg virus of the bunyaviridae family, targets the nucleolus and triggers nucleolar disruption [34], and redistribution of UBF, Nucleolin and NPM1 during adenovirus infection promotes nuclear reorganization, viral DNA replication, and virion assembly [24,35,36,37,38,39,40]. An almost complete destruction of the nucleolar structure, involving dispersion of nucleolar proteins, including UBF, Fibrillarin, Nucleolin and NPM1 to distinct cellular sites, has been described following infection with the herpes simplex virus type 1 (HSV-1) [41,42]. Redistribution of the nucleolar proteins following HSV-1 is partially dependent on the conserved late viral protein, UL24 [43,44,45]. Yet, it has been suggested that rRNA transcription and synthesis of ribosomal proteins, along with assembly of ribosomes, continue at a rate only slightly below that in uninfected cells [46,47]. Nevertheless, an altered rRNA maturation pathway has been described in HSV-1-infected cells [48]. Significantly, nucleolar proteins were found in proteomes that associate with viral genomes during infection with Adenovirus and HSV-1 [49].

Three cellular nucleolar proteins, including Angiogenin, NPM1 and Nucleolin, were shown to regulate KSHV infection, and three viral lytic gene products, ORF57, vBcl-2 and ORF20, localize to the nucleolus. Specifically, the infection of endothelial cells with KSHV induces the expression and secretion of the angiogenesis factor Angiogenin, which in turn translocates into the nucleus and to the nucleolus of subconfluent cells, stimulates proliferation and 45S rRNA gene transcription, and inhibits apoptosis [50]. vCyclin-Cdk6-mediated phosphorylation of NPM activates its association with LANA-1 to facilitate the interaction of LANA-1 with HDAC1 and core histones to sustain latency, while NPM knock-down leads to viral reactivation [51]. The binding of Nucleolin to IL-6 mRNA 3′ UTR was shown to promote escape from virus-induced degradation of host mRNAs [52]. ORF57 has major roles in transcriptional activation, RNA stability, RNA nuclear export and translational enhancement [53,54]. vBcl-2 is a homolog of the Bcl-2 family of apoptosis and autophagy regulators [55,56,57], and targets the nucleolus via interaction with the nucleolar protein PICT-1 [58]. ORF20 is a member of the UL24 protein family, which is conserved in all three subfamilies of the Herpesviridae. ORF20 co-purifies with 40S and 60S ribosomal subunits, and is associated with polysomes [59], however, it is not yet known whether the association of ORF20 with ribosomal proteins is established in the nucleolus during ribosomal biogenesis or at a later time point.

In the present study, we describe alterations in the nucleolar organization during lytic reactivation of KSHV-infected cells, involving redistribution of nucleolar proteins which are known to be involved in the biogenesis of the ribosomal subunits. Yet, we provide evidence that pre-rRNA transcription proceeds and rRNA processing continues. Finally, we demonstrate small changes in rRNA modifications (pseudouridylation and 2′-*O*-methylation) upon lytic virus reactivation, likely affecting ribosome function during virus propagation.

## 2. Materials and Methods

### 2.1. Cell Culture and Viruses

Human renal cell carcinoma SLK and iSLK cells (kindly provided by Don Ganem, Howard Hughes Medical Institute, UCSF, San Francisco, CA, USA and Rolf Renne, University of Florida, Gainesville, FL, USA) [60] were grown in Dulbecco’s modified Eagle’s medium (DMEM) (Biological Industries, Beit Haemek, Israel) containing 50 IU/mL penicillin and 50 µg/mL streptomycin (Biological Industries, Beit Haemek, Israel), and supplemented with 10% heat-inactivated fetal calf serum (FCS) (Biological Industries, Beit Haemek, Israel). iSLK cells were grown in the presence of 250 µg/mL G418 and 1 µg/mL Puromycin (A.G. Scientific Inc., San Diego, CA, USA) to maintain the Tet-on transactivator and the RTA expression cassette, respectively. iSLK cells were infected with BAC16 [61], BAC16-mCherry-ORF45 [62] or BAC16-Blue fluorescent protein (BFP)-mCherry in which GFP has been replaced by BFP (unpublished). The growth medium of infected iSLK cells was supplemented with 600 µg/mL hygromycin (MegaPharm, San Diego, CA, USA). KSHV-infected iSLK cells were treated with 1 μg/mL Dox and 1 mM sodium butyrate (Sigma, Santa Cruz, CA, USA), in the absence of hygromycin, puromycin, and G418, to induce RTA transgene expression and lytic cycle reactivation. Then, 0.5 μM Phosphonoacetic acid (PAA) (Sigma) was added to inhibit viral DNA replication. TREx BCBL-1 Rta-inducible cells [63] were cultured in RPMI1640 (Biological Industries, Beit Haemek, Israel) supplemented with 10% FCS and 200 µg/mL hygromycin and treated with 1 μg/mL Dox to induce the viral lytic cycle.

### 2.2. Fluorescence and Immunofluorescence Assay (IFA)

Cells were seeded on coverslips in a 24-well plate, washed with PBS (Biological Industries, Israel), and fixed with 4% formaldehyde in PBS for 25 min at room temperature. After fixation, cells were washed with PBS, and permeabilized and blocked in PBS containing 0.2% Triton X-100% and 1% BSA. Slides were incubated with primary antibodies to UBF (Sigma/Santa Cruz, CA, USA), Fibrillarin (Abcam, Cambridge, UK), NPM (Abcam, Cambridge, UK), Nucleolin (Abcam, Cambridge, UK), RPA194 (Santa Cruz, CA, USA), PICT-1 (Santa Cruz, CA, USA), PML (Santa Cruz, CA, USA), ORF45 (Santa Cruz, CA, USA), ORF65 [64] (a kind gift from Shou Jiang Gao) or ORF59 (a kind gift from Prof. Bala Chandran, University of South Florida) [65] at 40 C, followed by incubation with secondary conjugated antibodies (Rhodamine, Cy3, Alexa Fluor 647 or Alexa Fluor 488, Jackson ImmunoResearch Laboratories, Inc., West Grove, PA) for 1 h at room temperature. To stain the nuclei, cells were incubated for 10-min with 0.05 µg/mL Hoechst dye (Sigma) in PBS. The slides were mounted with anti-fading medium (1% n-Propyl gallate, 90% Glycerol in PBS). Cells were examined and photographed under a confocal laser-scanning microscope (Leica SP8 Confocal Live Microscope).

### 2.3. IFA Combined with Fluorescence In Situ Hybridization (FISH)

Cells were washed with PBS, mounted on poly-L-lysine-coated slides, and fixed with 1.6% formaldehyde in PBS at room temperature for 30 min. Cells were then treated with 1% Triton X-100 for 3 min, washed with PBS and prehybridized with hybridization buffer (60% deionized formamide, 50 mM sodium phosphate, pH 7.2, 0.5 mg/mL salmon sperm DNA, 1 µg/mL tRNA, and 5X Denhardt’s solution in 2X SSC) overnight at 720C. ITS-1 RNA probe was prepared by PCR using DNA from HeLa cells as template and forward and reverse primers 5′-CCTGTGGGGTGGTGTC-3′ and 5′-TTAATACGACTCACTATAGGGGTTGCCTCAGGCCG-3′, respectively. The resulting product containing a T7 promoter was cloned into pGEM-T Easy vector and was used to prepare anti-sense RNA probe labelled with biotin-UTP. The probes were diluted in 50% formamide, denatured for 5 min at 850C, and immediately chilled on ice. Next, probes were diluted in hybridization buffer, denatured again for 5 min at 850C, and immediately added to the slides at a final concentration of 2 ng per slide. The slides were covered, sealed, heated for 15 min at 850C, and transferred to 550C for overnight incubation. Slides were washed with 2X SSC for 5 min at room temperature, 0.2X SSC for 30 min at 60 °C, and finally with 0.2X SSC for 5 min at room temperature, and then incubated with Alexa Fluor 647 streptavidin conjugate (Molecular Probes). Slides were washed with 0.2X SSC and then incubated with 0.2X SSC containing 10% Bovine Serum Albumin at room temperature for 30 min. Then, cells were incubated antibodies to Fibrillarin for 2 h, washed with 0.2X SSC, and incubated for 1 h with secondary conjugated antibody (Rhodamine). Nuclei were stained with DAPI, and cells were visualized under Leica SP8 Confocal Live Microscope.

### 2.4. Reverse-Transcription (RT)-Quantitative PCR (RT-qPCR)

Total RNA was extracted by using EZ-RNA total RNA isolation kit (Biological Industries, Israel). Residual DNA contamination was eliminated by subsequent treatment with DNase (Turbo DNA-free kit, Ambion, Austin, TX, USA). cDNA was generated using 1 µg total RNA with a qScript cDNA synthesis kit (Quanta Biosciences, Gaithersburg, MD, USA) primed with random hexamers, according to the manufacturer’s instructions. RT-qPCR was performed in a total volume of 10 µL with 1.5 µL of cDNA diluted 1:2, and 0.15 M of each primer specific for rRNA, and Fast Sybr green master mix (Applied Biosystems, Waltham, MA, USA). The expression levels obtained for each gene were normalized to those of 18S RNA. The primers used were as follows: for 47S (rRNA), 5′-TGTCAGGCGTTCTCGTCTC-3′ and 5′-GAGAGCACGACGTCACCAC-3′; for 18S, 5′-ACCGATTGGATGGTTTAGTGAG-3′ and 5′-CCTACGGAAACCTTGTTACGAC-3′. All PCRs were run in triplicate on a StepOne Plus real-time PCR system (Applied Biosystems Inc., Carlsbad, CA, USA).

### 2.5. Northern Blot Hybridization

Total RNA was prepared with EZ-RNA total RNA isolation kit (Biological Industries, Israel), and 20 µg samples were loaded and fractionated on a 1.2% agarose, 2.2 M formaldehyde gel. The RNA was visualized with ethidium bromide. Small RNAs were analyzed on a 10% polyacrylamide gel containing 7 M urea. The RNA was transferred to a nylon membrane (Hybond; Amersham Biosciences) and probed with the following γ-32P-end-labeled oligonucleotides: 5.8S rRNA: 5′-TCAGACAGGCGTAGCCCCGGGAGGAACCCG-3′, 18S rRNA: 5′-ATCGGCCCGAGGTTATCTAGAGTCACCAAA-3′, and 28S rRNA: 5′-CCTCTTCGGGGGACGCGCGCGTGGCCCCGA-3′. For labeling, 50 pmoles of each probe was incubated with 50 pmoles of [γ32P]ATP and T4 polynucleotide kinase (Promega) for 30 min at 37 °C. In all cases, membranes were washed twice in 2X SSC, 0.1% SDS at 60 °C for 20 min, and once in 0.1X SSC, 0.1% SDS at 43 °C for 15 min. Then, 18S and 28S rRNA visualized by ethidium bromide was used as loading control.

### 2.6. Mapping the Pseudouridine Nucleotides by the CMC Method

Pseudouridine sites were mapped as previously described [66]. RNA from BAC16-mCherry-ORF45-infected iSLK cells and SLK cells that were treated with Dox and n-Butyrate for 48 h was extracted and treated with CMC (N-cyclohexyl-N′-β-(4-methylmorpholinium) ethylcarbodiimide p-tosylate) in CMC buffer (0.17 M CMC in 50 mM bicine, pH 8.3, 4 mM EDTA, 7 M urea) at 370 °C for 20 min. Under these conditions, the incorporation of CMC in the place of Ψ terminates reverse transcription one nucleotide 3′ to the pseudouridylated base, and thereby allows analysis of RNA pseudouridylation at single nucleotide resolution. To remove all the CMC groups except those linked to the Ψ, the CMC-treated RNA was subjected to alkali hydrolysis with Na_2_CO_3_ (50 mM, pH 10.4) at 37  °C for 4 h. The RNA was then fragmented to a size range of 50–150 nt, adaptor was ligated to the 3′ ends, and cDNA was prepared using reverse transcriptase. Then, an adaptor was ligated to the cDNA, and after amplification, the samples were sequenced in Illumina machine in paired end mode. The reads were mapped to human rRNA using Smalt v0.7.5 (default parameters). Each read pair was “virtually” extended to cover the area from the beginning of the first read to the end of its mate. For each base, the number of reads initializing at that location as well as the number of reads covering the position were calculated. A combination of Bedtools and in-house Perl scripts were used to calculate the Ψ-ratio and Ψ-fc (fold change). For each nucleotide, we computed the Ψ-ratio, dividing the number of reads covering that nucleotide by the number of nucleotides initiating at the following base (i.e., corresponding to the last position copied by the reverse transcriptase). This was repeated for (−CMC) and (+CMC) samples. The Ψ-fc was computed as the log2-fold change of the Ψ-ratios in iSLK versus the SLK samples. We applied this threshold to each sample. Primer extension was performed as previously described [67] with or without CMC and 5′-end-labeled oligonucleotides specific to target rRNAs: Ψ 4636–4628: 5′-CCCACAGATGGTAGCTTCGC-3′, Ψ 4689: 5′-GCCGTATCGTTCCGCCTGGG-3′, Ψ 4361: 5′-CAAGCCAGTTATCCCTGTGG-3′. The extension products were analyzed on 12% polyacrylamide–7 M urea gel, next to sequencing reactions performed using the same primer. Band intensity was quantified using ImageJ software (http://imagej.nih.gov/ij/, accessed on 16 July 2022).

### 2.7. Hydrazine and Aniline Treatment

Total RNA (5 µg) was treated with 50% hydrazine (Sigma) for 45 min on ice, and ethanol precipitated [68]. The RNA pellet was then resuspended in 1 M aniline (Sigma) (pH 4.5 adjusted using glacial acetic acid) until the white pellet was completely dissolved, boiled for 15 min at 60 °C in the dark, and immediately placed on ice. The fragmented RNA was recovered by ethanol precipitation, and used for library preparation [69].

### 2.8. HydraPsiSeq Library Preparation

To perform HydraPsiSeq, the fragmented RNA (~800 ng) was dephosphorylated with FastAP Thermosensitive Alkaline Phosphatase (Thermo Scientific, Waltham, MA, USA), cleaned by Agencourt RNA clean XP beads (Beckman Coulter, Brea, CA, USA) and ligated to a 3′ linker using high concentration T4 RNA Ligase 1 (NEB, Ipswich, MA, USA) in a buffer containing DMSO, ATP, PEG 8000, and RNase inhibitor (NEB, Ipswich, MA, USA). The ligated RNA was cleaned from excess linker using Dynabeads^®^ MyOne™ SILANE beads (Thermo Scientific, Waltham, MA, USA), and first strand cDNA was prepared using the AffinityScript Reverse Transcriptase (Agilent, Santa Clara, CA, USA). The RNA was subsequently degraded using 2 µL of 1 M NaOH, and the cDNA was cleaned using Dynabeads^®^ MyOne™ SILANE beads (Thermo Scientific, Waltham, MA, USA). The cDNA was further ligated to a 3′ adapter using a high concentration T4 RNA Ligase 1 (NEB, Ipswich, MA, USA) and cleaned of excess adapter using Dynabeads^®^ MyOne™ SILANE beads (Thermo Scientific, Waltham, MA, USA). The adapter-ligated cDNA was PCR enriched using NEBNext^®^ High-Fidelity (NEB, Ipswich, MA, USA) polymerase (9 PCR cycles), separated on an E-Gel EX agarose gel (Invitrogen, Waltham, MA, USA) and size selected at the range of 150–300 bp (containing ~30–180 nt corresponding to RNA). The amplicons were gel purified using NucleoSpin Gel and PCR Clean-up kit (Macherey-Nagel, Düren, Germany), and sequenced in a Nextseq system (Illumina, San Diego, CA, USA) in paired end mode (20 million reads for each sample).

### 2.9. HydraPsiSeq Data Analysis

The paired end reads obtained from each sample were aligned to the human rRNA sequence using Smalt v_0.7.5 (http://www.sanger.ac.uk/resources/software/SMALT/, accessed on 16 July 2022) with default parameters. For each sample, the resulting bam file was sorted and filtered for proper pairs using Samtools v1.9 [70] and then converted to a BED file using the bamtobed module from the BEDtools v2.26.0 Suite [71]. Using an in-house Perl script on each bed file, the number of reads whose 5’-end alignments initiate at that base for each position on the rRNA was calculated. The total coverage for each base was then determined using the genomecov module from the BEDtools v2.26.0 Suite [71]. These files were then used as input for the R scripts (https://github.com/FlorianPichot/HydraPsiSeqPipeline, accessed on 16 July 2022), as previously described [68].

### 2.10. Alkaline Hydrolysis and RiboMeth-Seq Library Preparation

Total RNA (5 mg) was denatured at 90 °C for 2 min in a thermocycler. Then, an equal volume of buffer (NaHCO_3_/Na_2_CO_3_, pH 9.9) was added, and RNA samples were incubated at 90 °C for 20 min (Birkedal et al., 2015). The hydrolyzed RNA was then used for library preparation. Briefly, 800 ng of the RNA was dephosphorylated with FastAP Thermosensitive Alkaline Phosphatase (Thermo Scientific, Waltham, MA, USA) and cleaned by Agencourt RNA clean XP beads (Beckman Coulter, Brea, CA, USA). The RNA was then ligated to 3′ linker using high concentration T4 RNA Ligase 1 (NEB, Ipswich, MA, USA) in a buffer containing DMSO, ATP, PEG 8000 and RNase inhibitor (NEB, Ipswich, MA, USA) for 1.5 h at 22 °C. The ligated RNA was purified from excess linker using Dynabeads^®^ MyOne™ SILANE beads (Thermo Scientific, Waltham, MA, USA), and first strand cDNA was prepared using the AffinityScript Reverse Transcriptase enzyme (Agilent, Santa Clara, CA, USA) at 55 °C for 45 min. Next, the RNA was degraded using 2 µL of 1 M NaOH, and the cDNA was cleaned using Dynabeads^®^ MyOne™ SILANE beads (Thermo Scientific, Waltham, MA, USA). The cDNA was further ligated to 3′ adapter using a high concentration T4 RNA Ligase 1 (NEB, Ipswich, MA, USA) overnight at 22 °C and cleaned of excess adapter by using Dynabeads^®^ MyOne™ SILANE beads (Thermo Scientific, Waltham, MA, USA). The adapter ligated cDNA was PCR enriched using NEBNext^®^ High-Fidelity (NEB, Ipswich, MA, USA) polymerase (9 PCR cycles), separated on an E-Gel EX agarose gel (Invitrogen, Waltham, MA, USA) and size selected for the range of 150–300 bp (containing ~30–180 nt corresponding to RNA). The amplicons were gel purified using NucleoSpin Gel and PCR Clean-up kit (Macherey-Nagel, Düren, Germany) and sequenced in a Nextseq system (Illumina, San Diego, CA, USA) in paired end mode (20–40 million reads for each sample).

### 2.11. RiboMeth-Seq Data Analysis

To analyze the RiboMeth-seq libraries, the reads were initially trimmed of adapter sequences using Trim Galore version 0.4.4 (https://github.com/FelixKrueger/TrimGalore, accessed on 16 July 2022) with the following parameters: --stringency 4 --length 30 --paired --retain_unpaired. The alignment to the reference rRNA sequence was performed by STAR (ver 2.0.6) (https://github.com/alexdobin/STAR/releases, accessed on 16 July 2022) [72], mapped, and properly paired reads were converted to *.bed using BEDtools v2.26.0 Suite [71]. Counting of 5′- and 3′-ends was performed directly on *.bed file using a dedicated Unix script. Analysis was performed by calculation of MAX score for detection of Nm residues and RMS score (score C) for their quantification. To calculate MAX score, the relative change of end coverage position by position was calculated in 5′→3′ and reverse direction. The relative change was normalized to average values spanning −6 and +6 nucleotides. The normalized relative change for 5′→3′ and reverse direction were averaged, and the maximal value between the average and normalized relative change was retained (score MAX). RMS score was calculated essentially as described previously for score C using the same relative impact of neighboring nucleotides [73].

## 3. Results

### 3.1. Lytic Reactivation of KSHV Is Associated with Major Changes in the Nuclear Organization and Redistribution of Nucleolar Proteins

The lytic cycle of KSHV involves the formation of nuclear replication compartments along with changes in nuclear structures [74]. To characterize these changes, we employed the human renal cell carcinoma SLK cell line and its derivative, iSLK, as model systems [60]. iSLK cells are widely used to study KSHV, as they can stably maintain latent infection and harbor a doxycycline (Dox)-inducible cassette of the KSHV gene product RTA/ORF50, which enables an efficient switch of the lytic cycle when treated with n-Butyrate (n-But). iSLK cells were infected with the recombinant bacterial artificial chromosome (BAC) KSHV clone, BAC16, which constitutively expresses GFP from the elongation factor 1-α-promoter [61], or with BAC16-mCherry-ORF45 clone which also express the immediate-early lytic gene product ORF45 fused to monomeric Cherry fluorescent protein (mCherry), and thereby allows tracking of cells undergoing lytic replication [62].

**Figure 1:**
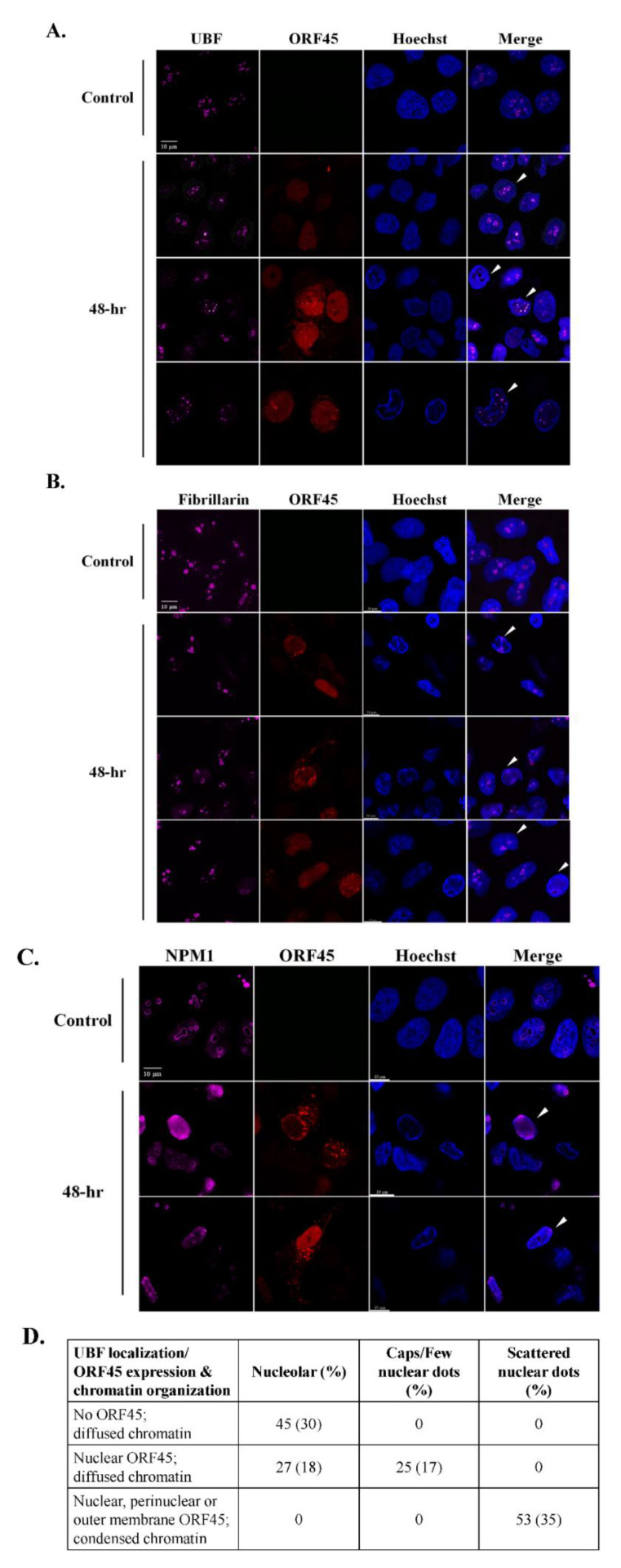
Redistribution of the nucleolar proteins UBF, Fibrillarin and Nucleophosmin 1 (NPM1) upon lytic reactivation of KSHV in iSLK-infected cells. BAC16-mCherry-ORF45-infected iSLK cells were treated for 48 h with 1 µg/mL Dox and 1 mM n-Butyrate to induce viral lytic reactivation. Uninduced infected iSLK cells were used as controls. Cells were stained with antibodies to UBF or (**A**), Fibrillarin followed by anti-rabbit Alexa Fluor 647-conjugated secondary antibody (**B**) or NPM1 followed by anti-mouse Alexa Fluor 647-conjugated antibodies (**C**). The corresponding staining of nuclear DNA by Hoechst is also shown. mCherry-ORF45 expression and localization and the appearance of condensed chromatin staining marked different stages of cells undergoing viral lytic reactivation. Arrows indicate different patterns of redistribution. Right-hand columns show the merged output of the nucleolar proteins immunofluorescence and Hoechst staining 150 cells were scored according to the cellular distribution of UBF along with ORF45 expression and localization and chromatin organization (**D**).

To observe the nucleolar organization during the productive cycle of KSHV, we treated BAC16- mCherry-ORF45-infected iSLK cells with Dox and n-But to induce lytic reactivation. Controls included uninfected SLK cells that were treated similarly which ruled out potential effects of chemical treatments (Supplementary Figure S1) as well as uninduced iSLK cells that were not treated with Dox and n-But. As in other experimental cell models of KSHV [75,76,77], the induction of lytic reactivation in infected iSLK cells is fractionally and asynchronous, and therefore, when examining cells post induction, we observed cells that did not undergo viral lytic reactivation as well as cells at different stages of the lytic cycle. These stages were distinguished by the intensity of the expression and the distribution of the lytic viral protein ORF45, which is predominantly nuclear at early lytic stages and perinuclear, cytoplasmic and close to the plasma membrane at later stages [62]. In addition, late stages of the lytic cycle are characterized by the formation of a large central nuclear zone devoid of chromatin, along with condensed chromatin at the nuclear periphery [9]. Cells were processed 48 h following lytic induction, by which time a significant proportion of the cells undergo lytic reactivation and DNA replication is well under way; however, uninduced cells and cells at early stages of the lytic cycle are also evident at this time point. Cells were stained with antibodies to UBF (Figure 1A), Fibrillarin (Figure 1B) and Nucleophosmin 1 (NPM1) (Figure 1C) which are commonly used to mark the FC, DFC and GC nucleolar substructures, respectively. It was determined through the nuclear expression of ORF45 and regular diffuse host chromatin distribution that, at early lytic stages, the rRNA transcription factor UBF, which is normally associated with rDNA at the rRNA promoters and plays a role in the recruitment of RNA pol I, remained in the nucleolar structures or redistributed into cap-like structures or into few dot-like structures in the nucleoplasm. At later stages, determined through the localization of ORF45 and chromatin condensation to the nuclear periphery, UBF presented scattered dot-like structures. No dispersion of the nucleolar protein Fibrillarin, which is the core protein of C/D small nucleolar RNAs (snoRNAs) that functions as 2′-*O*-methytransferase and is involved both in methylation and processing of rRNA, was evident in cells expressing nuclear ORF45 with intact diffused chromatin. Yet, at later stages of the lytic cycle, this protein was localized within 1–3 large peripheral foci surrounded by dense chromatin reminiscent of the nucleolar compartment. NPM1, a multifunctional nucleolar protein that normally resides in the GC substructure, exhibited two major patterns upon induction of the lytic cycle, characterized either by diffuse nuclear staining or marginal nucleolar staining. Similar patterns of UBF and Fibrillarin distribution were documented upon lytic reactivation in iSLK BAC16-infected cells in which the expression of ORF45 was detected with antibodies (Supplementary Figure S2), as well as in the PEL-derived cell line BCBL-1 in which the expression of the small capsid protein ORF65 was used to identify cells undergoing lytic replication (Supplementary Figure S3).

### 3.2. The Nucleolar Protein UBF Does Not Colocalize with Promyelocytic Leukemia (PML) Nuclear Bodies

**Figure 2:**
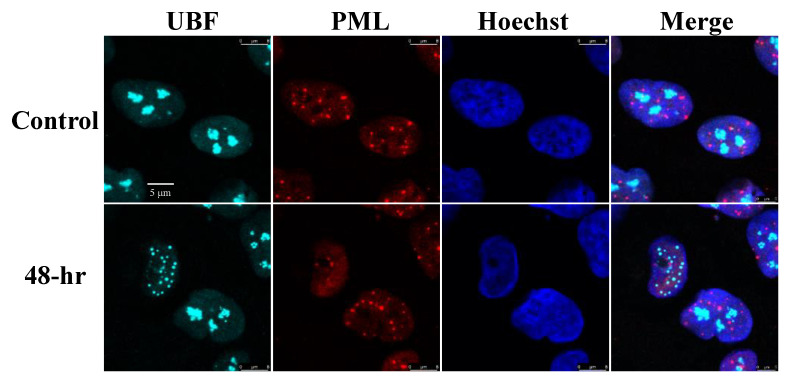
UBF does not colocalize with PML bodies. BAC16-infected iSLK cells were treated for 48 h with 1 µg/mL Dox and 1 mM n-Butyrate to induce lytic reactivation. Uninduced BAC16-infected iSLK cells were used as a control. Cells were stained with anti-PML followed by Rhodamine-conjugated secondary antibody. Subsequently, the cells were incubated with antibodies to UBF, and then with anti-rabbit Cy5 secondary antibody. Chromatin was detected by Hoechst staining.

PML bodies are subnuclear structures involved in a wide range of cellular functions, including transcription control, protein modifications, DNA damage response, apoptosis, senescence and antiviral responses [78]. Given the known functions of the PML bodies during viral infections and in view of their well-known dot-like nuclear distribution, we examined potential colocalization of UBF with PML bodies during lytic reactivation. As shown in Figure 2, in line with previous reports [79,80] a decreased number of PML bodies was evident in cells undergoing lytic replication identified by condensed chromatin at the nuclear periphery. However, UBF was not colocalized with foci of PML nuclear bodies during the latent or the lytic cycle of KSHV infection.

### 3.3. Redistribution of UBF and Fibrillarin Depend on Viral DNA Replication or Late Viral Gene Expression

**Figure 3:**
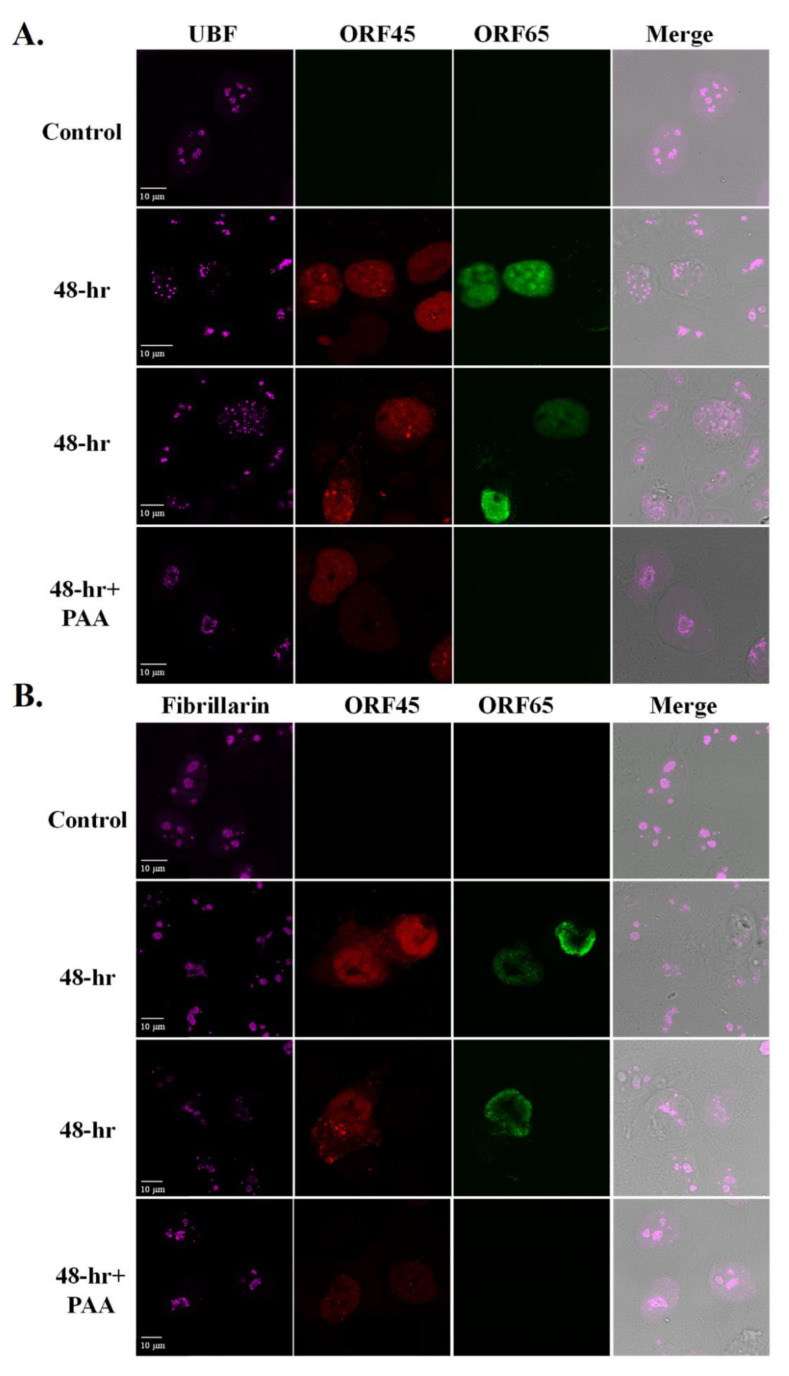
Redistribution of UBF and Fibrillarin depends on viral DNA replication. BAC16-BFP-mCherry-infected iSLK cells were treated for 48 h with 1µg/mL Dox and 1 mM n-Butyrate to induce lytic reactivation. Treatment with PAA was carried 2 h prior to lytic induction and continued during lytic reactivation. Infected iSLK cells that were either left untreated or induced to undergo lytic reactivation with no PAA treatment were used as control. mCherry-ORF45 was used to track cells undergoing lytic reactivation while ORF65, detected with anti-ORF65 antibody followed by anti-mouse Alexa Fluor 488, was used to distinguish late viral lytic stage and effectiveness of PAA treatment. Cells were then stained with anti-UBF (**A**), or anti-Fibrillarin (**B**) followed by anti-rabbit Alexa Fluor 647-conjugated secondary antibody. Right-hand columns show the merged output of the nucleolar proteins immunofluorescence with differential interference contrast (DIC) microscopy images.

The redistribution of Nucleolin and NPM1 during HSV-1 infection was previously shown to depend on the expression of the late viral protein UL24 [43,44,45,81]. Accordingly, we examined whether treatment with the viral DNA polymerase inhibitor phosphonoacetic acid (PAA), which inhibits virus replication and expression of late viral genes, would alter UBF and Fibrillarin localization during lytic reactivation of KSHV [82]. iSLK cells that carry latent infection with BAC16-Blue fluorescent protein (BFP)-mCherryORF45, which constitutively expresses BFP and expresses ORF45 upon lytic reactivation, were induced to undergo lytic reactivation for 48 h in the absence or presence of PAA. As shown in Figure 3 and in line with previous reports [82], treatment with PAA reduced the expression of the immediate-early lytic ORF45 protein and abolished the expression of the late small capsid protein ORF65. Redistribution of UBF into cap-like structures with no nuclear dots was evident, while Fibrillarin remained within nucleolar-like structures upon PAA treatment. This implies that the redistribution of these proteins during lytic reactivation depends on viral DNA replication, on the formation of viral replication compartments, or on late viral gene products that are not expressed under PAA treatment.

### 3.4. UBF Is Partially and Transiently Colocalized with Replication Compartments, Whereas Fibrillarin Does Not Colocalize with Replication Compartments during Lytic Induction

**Figure 4:**
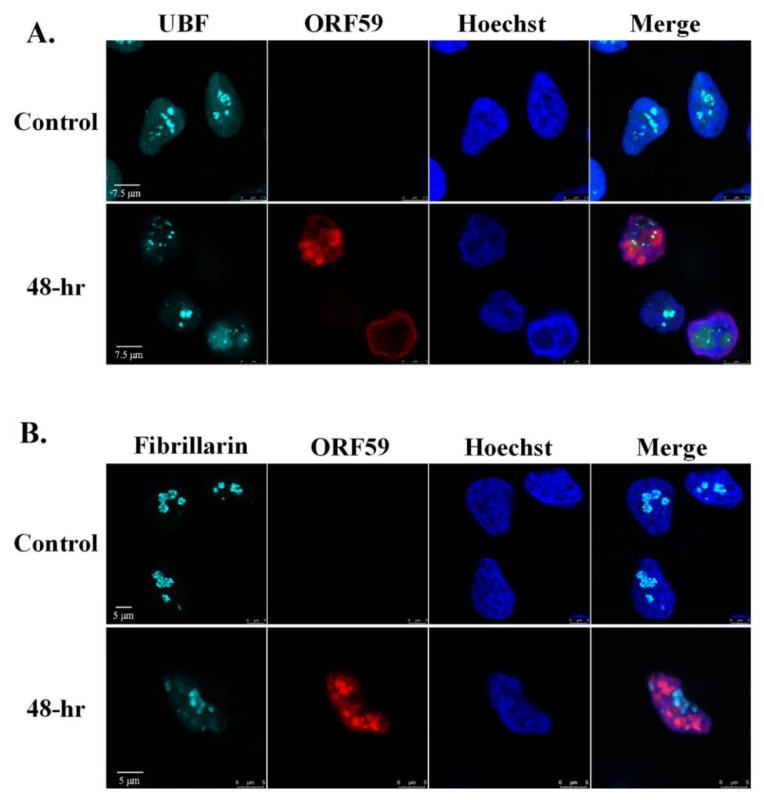
UBF partially colocalizes with viral replication compartments whereas Fibrillarin does not colocalize with replication compartments during lytic reactivation. BAC16-infected iSLK cells were treated for 48 h with 1µg/mL Dox and 1 mM n-Butyrate to induce lytic reactivation. Untreated infected iSLK cells were used as control. Cells were stained with anti-ORF59 and secondary Rhodamine-conjugated antibody. Cells were subsequently incubated with anti-UBF (**A**) or anti-Fibrillarin (**B**) and then incubated with anti-rabbit Cy5-conjugated secondary antibody. Chromatin was detected by Hoechst staining.

Next, we examined the cellular localization of UBF and Fibrillarin along with ORF59 viral protein which functions as a processivity factor during viral DNA replication, and hence is localized in the viral replication compartments during the lytic cycle [65,83,84]. As shown in Figure 4, and in Movies S1 and S2, UBF was clearly observed within the replication compartments and UBF foci overlapped with ORF59. In contrast, Fibrillarin did not share common localization with ORF59. Rather, ORF59 occupied most of the nuclear zone whereas Fibrillarin appeared in large chromatin enclosed structures.

### 3.5. Redistribution Pattern of UBF during Lytic Reactivation Overlaps with the Nucleolar Proteins RPA194 and PICT-1 but Not with Fibrillarin

**Figure 5:**
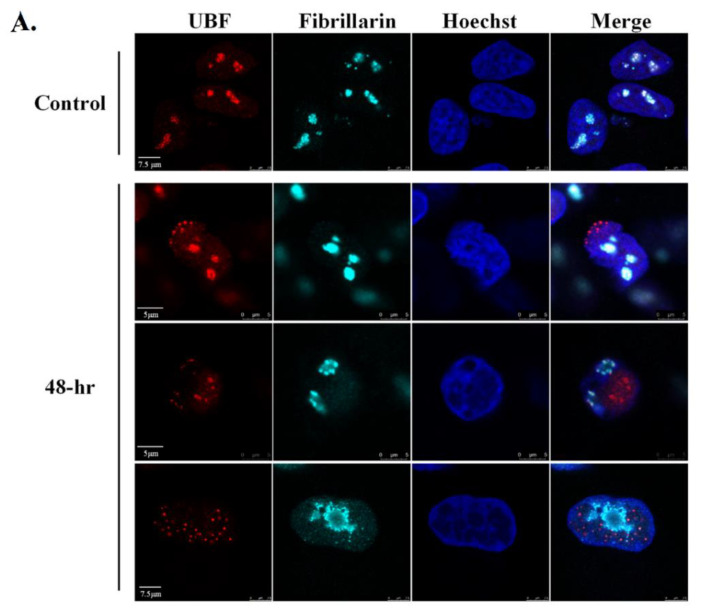

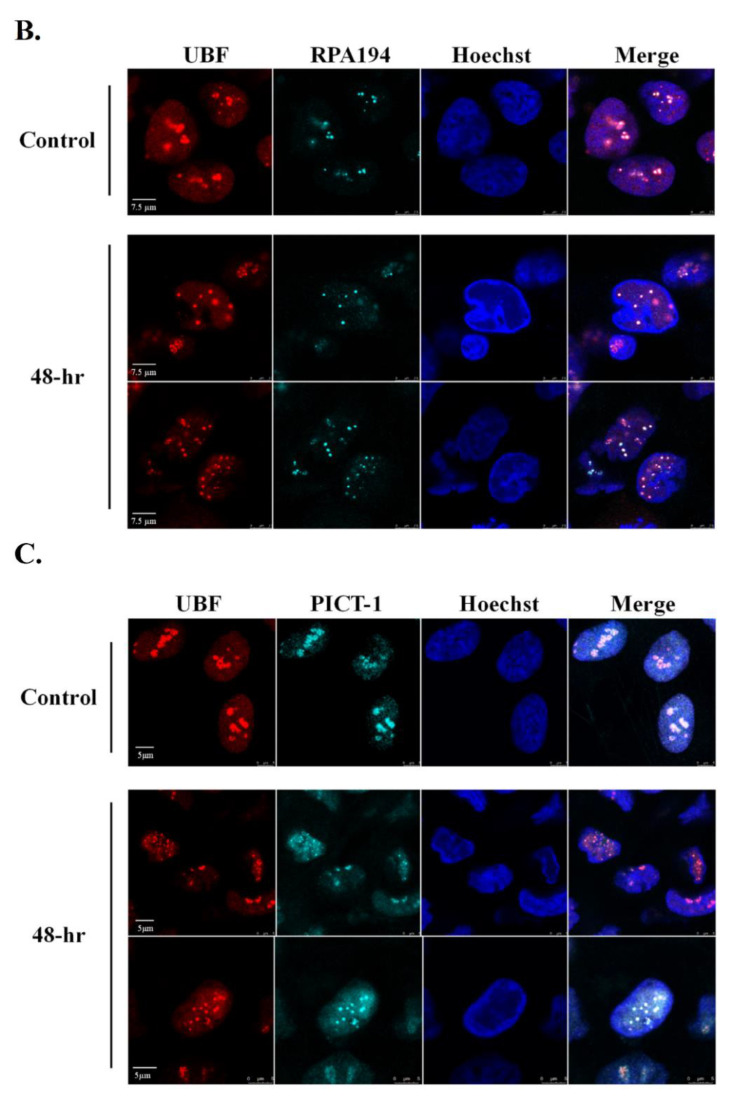
UBF shares common redistribution kinetics and pattern with RPA195 and PICT-1 but not with Fibrillarin during lytic reactivation. BAC16-infected iSLK cells were treated for 48 h with 1µg/mL Dox and 1 mM n-Butyrate to induce lytic reactivation. Uninduced infected iSLK cells were used as controls. Cells were stained with mouse anti-UBF and Rhodamine-conjugated secondary antibody. Cells were then incubated with anti-Fibrillarin, which was detected by anti-rabbit Cy5-conjugated secondary antibody (**A**). Cells were stained with rabbit anti-UBF and Cy3-conjugated secondary antibody, and then with anti-RPA194 (**B**) or anti-PICT-1 (**C**), which were detected by anti-mouse Cy5-conjugated secondary antibody. Chromatin was detected by Hoechst staining.

As UBF and Fibrillarin appear to exhibit different localization patterns after lytic reactivation, we next examined their cellular localization at the same cells and with additional nucleolar proteins as well. As shown in Figure 5A and Movie S3, UBF and Fibrillarin demonstrated different dispersion patterns with respect to each other. At early activation stages, when the chromatin distribution was still normal, UBF already began to disperse, though most of it was still found in the nucleoli. At this stage, Fibrillarin remained within large nuclear foci that likely represented the nucleoli (Figure 5A, second row). At more advanced lytic reactivation stages, there was little overlap between UBF, which appeared to be scattered throughout the nucleoplasm, and Fibrillarin which was found mainly within an enclosed nuclear body, or bodies, at the periphery. In contrast, UBF and the large RNA polymerase I subunit RPA194 shared similar redistribution kinetics and pattern after lytic reactivation in all cells examined (Figure 5B). Similarly, the nucleolar exosome recruiting and assembly factor, PICT-1 [85,86] also shared redistribution kinetics and patterns with UBF (Figure 5C). Finally, partial colocalization was evident between UBF, which formed small dots, and Nucleolin, which redistributed to the nucleoplasm, probably within replication compartments (Movie S4). These findings suggest that the dispersion of nucleolar proteins during lytic reactivation is regulated. Proteins that are likely to share common roles remain co-localized, whereas other proteins disperse to different locations and hence may have distinct functions. Furthermore, the common cellular distribution of the rRNA transcription factor UBF, the RNA polymerase I subunit RPA194, and PICT-1 suggests that rRNA transcription in these cells is maintained.

### 3.6. rRNA Transcription and Processing Persist during Lytic Reactivation of KSHV

**Figure 6:**
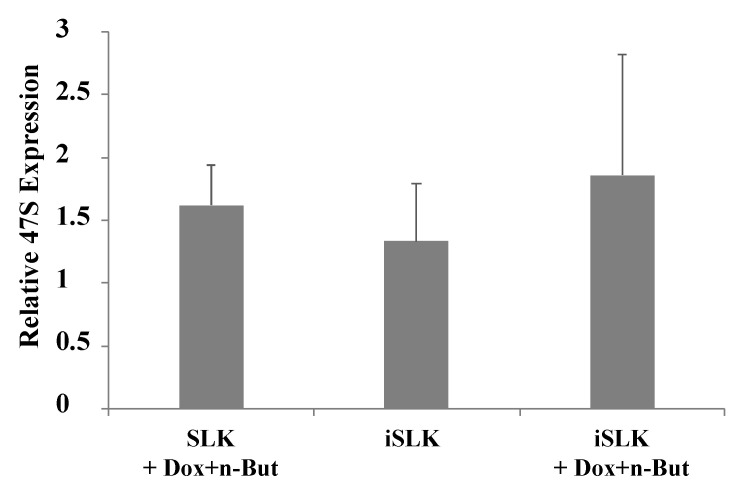
Lytic reactivation does not alter expression levels of the 47S pre-rRNA. Uninfected SLK and BAC16-mCherry-ORF45-infected iSLK cells were treated for 48 h with 1 µg/mL Dox and 1 mM n-Butyrate to induce lytic reactivation. Untreated cells were used as controls. Lytic reactivation was confirmed by expression of mCherry-ORF45 in ~80% of cells. The 47S pre-rRNA levels were determined using RT-qPCR with primers that target the 5′ external transcribed spacer (ETS) of pre-rRNA, and the results obtained were normalized to 18S rRNA. Data are presented relative to untreated SLK cells, and are from the averages of five biological repeats, each including three technical replicates. Error bars represent standard deviations. No statistically significant differences between SLK and iSLK cells prior or after lytic reactivation were obtained (one-way ANOVA and one-sample *t*-test).

In mammalian cells, the initial transcription product of rDNA is known as the 47S pre-rRNA. Processing of this transcript involves a series of cleavages and chemical modifications and results in the production of intermediate rRNAs, including 45S, 41S and 32S rRNA, and the three mature rRNAs: the 28S, 18S and 5.8S species. Given the changes in the nuclear organization and in the distribution of key nucleolar proteins, we aimed to characterize changes in the synthesis, localization and processing of the rRNA. First, we quantified pre-rRNA levels using RT-qPCR with primers that target the 5′-external transcribed spacer (5′-ETS) which is included in the 47S pre-rRNA transcript but not in the mature rRNA. This analysis employed BAC16-mCherry-ORF45-infected iSLK cells which enabled tracking lytic reactivation in ~70% of cells. As shown in Figure 6, no significant changes in the levels of pre-rRNA were detected upon lytic induction, suggesting slow degradation or continuous transcription of pre-rRNA during productive KSHV infection.

**Figure 7:**
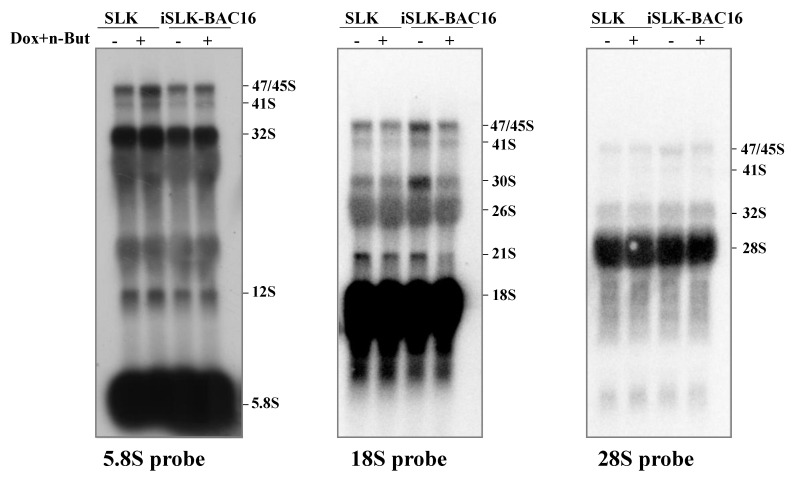
Northern blot analysis of rRNA during viral lytic reactivation. Total RNA was extracted from uninfected SLK and BAC16-mCherry-ORF45-infected iSLK cells that were either left untreated or treated with Dox and n-Butyrate for 48 h. Lytic reactivation was confirmed by the expression of mCherry-ORF45 in ~70% of cells. A total of 20 µg RNA from each sample was resolved on denaturing 1.2% agarose gel, transferred to nylon membranes, and hybridized with probes representing 5.8S, 18S and 28S rRNA. No accumulation of pre-rRNA processing products was evident upon lytic reactivation.

**Figure 8:**
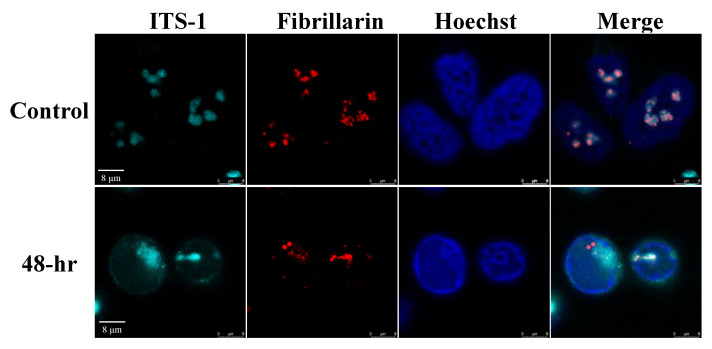
RNA in situ hybridization analysis to determine the location of the pre-rRNA transcripts and Fibrillarin in cells during viral lytic reactivation. SLK and BAC16-infected iSLK cells were treated with 1µg/mL Dox and 1 mM n-Butyrate. Cells were incubated with ITS-1 probe and stained with anti-Fibrillarin and Cy3-conjugated secondary antibody. Hoechst staining was used to detect chromatin.

In addition, no accumulation of the selected pre-rRNA intermediates was found by Northern blot analysis, suggesting that the proper processing of pre-rRNA continued (Figure 7). Significantly, unique rRNA processing products were previously identified in HSV-1 infected cells during lytic infection [48]. However, no unique products were identified here, and no significant differences in the relative expression levels of the different processing products were detected. As the nucleolar protein Fibrillarin is known to participate in the processing of pre-rRNA, we examined the cellular localization of this protein along with the relatively long-lived internal transcribed spacer 1 (ITS-1) pre-rRNA sequence by using FISH combined with immunofluorescence. As shown in Figure 8, in control cells, the ITS-1 fluorescent signal accumulated mainly in nucleoli and colocalized with Fibrillarin. However, during lytic reactivation, as identified through the marginalization of the nucleolar structure and by low-density chromatin, the ITS-1 was detected as dots, and mostly overlapped with Fibrillarin, suggesting that the processing of rRNA continues during lytic reactivation.

Although the possibility that changes in the half-life of mature rRNAs during lytic reactivation has not been ruled out, when taken together, the lack of accumulation of selected pre-rRNA intermediates suggests that rRNA transcription and processing proceed during lytic reactivation of KSHV, despite the different distribution of nucleolar proteins, which are known to participate in these processes.

### 3.7. Changes in Pseudouridine and 2′-O-Methylation Levels at Specific rRNA Sites

Recently, it was postulated that rRNA modifications may provide an important source of ribosome heterogeneity, and tune ribosome function in response to various signals [87,88,89,90,91,92]. These modifications, which may be fractional at certain positions, stabilize the secondary and tertiary structure of the rRNA scaffold, thereby modulating the efficiency and accuracy of protein translation. One of the most abundant rRNA modifications in eukaryotes involves the isomerization of uridine to pseudouridine (Ψ). To identify Ψ sites which change during lytic reactivation, we initially mapped the Ψ across the rRNA based upon CMC (N-cyclohexyl-N’-β-(4-methylmorpholinium) ethylcarbodiimide p-tosylate) modification followed by alkaline treatment. Under these conditions, the reaction of Ψ sites with CMC results in inefficient reverse transcription during the library preparation process, with the reverse transcription product terminating one nucleotide before the modified base [93]. We prepared RNA-seq libraries from total RNA from BAC16-infected iSLK and control SLK cells that were treated with Dox and n-But to induce lytic reactivation with and without CMC treatment. The ratio of the number of reads supporting reverse transcriptase termination to the number of overlapping reads (known as the Ψ-ratio) was calculated [94]. The Ψ-fold change (Ψ-fc) is the log2-transformed Ψ-ratio of the treated samples (+CMC) divided by the Ψ-ratio in the non-treated samples (−CMC). Comparing the Ψ-fc across replicates identified changes in Ψ levels at selected positions, illustrated in Supplementary Figure S4A and detailed in Supplementary Table S1. The most conspicuous changes observed were the increase in modification (hyper-modifications) at six positions—two located in domain IV (H69; LSU_ Ψ3695 and Ψ3758), one in domain VI (LSU_ Ψ4973), and two in or around the peptidyl transferase center (PTC; LSU_ Ψ4361 and Ψ4457). Hypomodified positions were identified in domain VI (LSU_ Ψ 4636 and Ψ 4689) and one in the PTC (LSU_ Ψ4521) (Figure S4B). Selected changes were further verified by primer extension (Figure S4C).

To obtain an independent assessment of the Ψ level, we utilized HydraPsiSeq which relies on the random cleavage of uridine nucleotides upon treatment with hydrazine and aniline. The ability of Ψ sites to resist fragmentation enables stoichiometric quantification of this RNA modification [68]. This method is considered more quantitative for assessing levels of Ψ modification. To this end, we calculated the PsiScore, that considers the total number of reads and 5′/3′ end coverage in each uridine nucleotide to estimate the faction of Ψ in each uridine nucleotide (Supplementary Table S2). Notably, using HydraPsi-seq we determined four variable Ψ sites (Ψ 3884, Ψ 4636 and Ψ 4973 in LUS, and Ψ 406 in SSU). Comparing differential level of Ψ using Ψ-seq and HydraPsiSeq, we observed that Ψ 4973 in LSU domain VI was hypermodified during viral reactivation by both the methods used. In addition, HydraPsiSeq detected hypomodified Ψ3884, located around the PTC, in cells undergoing lytic reactivation. It is currently unknown why the level of some Ψ sites vary between the two methods. Yet, it is well known that reverse transcriptase-dependent methods, such as Ψ-seq are prone to noise due to secondary structures within the RNA molecule [95], thus the differential levels of modification using Ψ-seq may reflect changes in rRNA structure as a result of viral replication. Further studies should support this idea.

**Figure 9:**
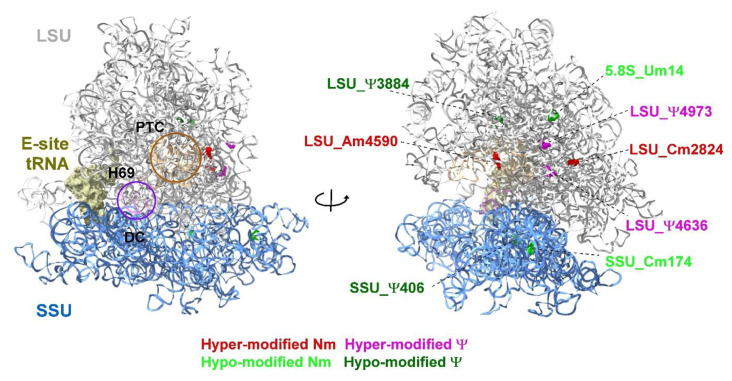
Localization of Ψ and Nm residues altered in KSHV-infected iSLK cells undergoing lytic reactivation. Uninfected SLK cells and BAC16-infected iSLK cells were treated with Dox and n-Butyrate for 48 h. The data are derived from HydraPsiSseq and RiboMeth-seq analysis. The location of peptidyl transferase center (PTC), E site tRNA, decoding center (DC) and helix H69 are shown. The large subunit (LSU) and small subunit (SSU) are colored in grey and blue, respectively. The 3D representation of human ribosome is based on the published CryoEM structure (PDB 6EK0) [98]. Figures were generated using UCSF Chimera-X software (https://www.rbvi.ucsf.edu/chimerax, accessed on 16 July 2022).

To gain insight into the level of 2′-*O*-methylation (Nm), which is the second most abundant RNA modification, we sought to analyze the stoichiometry of Nms using RiboMeth-seq [69,73]. In this method, the methylated nucleotides are resistant to alkaline hydrolysis and this property, combined with deep sequencing, allows to estimate the stoichiometry of the Nm residue. The fraction of methylated nucleotide (Score C or RMS) is calculated by profiling the coverage of nucleotides upon alkaline hydrolysis [69,73]. Using this method, we identified two hyper (LSU_Cm2824 and LSU_Am4590) and two hypo-modified (5.8S_Um14 and SSU_Cm174) Nm residues that change upon lytic viral reactivation. Localization of the hypo and hyper-modified Ψ (by HydraPsiSeq) and Nm sites on a 3D structure of the human ribosome suggests that all the modifications in functional domains, such as PTC/entrance tunnel, H69, and decoding center are not altered during lytic virus replication (Figure 9 and Supplementary Tables S2 and S3). Notably, all eight modifications that were found to be altered by quantitative mapping (HydraPsiSeq and RiboMeth-seq) are partially modified sites [96] and are not located within functional domains. These results are reminiscent of changes observed in cancer cells, whereby increases and decreases in distinct Ψ and Nm positions were observed outside the rRNA functional domains [97].

## 4. Discussion

The protein content and organization of the nucleolus are dynamic and change under different stress conditions, such as transcriptional inhibition and DNA damage [98]. Major alterations in the morphology and composition of the nucleoli have been previously reported following HSV-1 infection [41,43,44]. Nevertheless, the persistence of rRNA transcription and ribosome biogenesis involving modified rRNA maturation through late stages of HSV-1 infection has been documented [46,48]. However, almost no description of the nuclear and the nucleolar morphology during KSHV infection is available to date.

By using immunofluorescence microscopy, we documented an extensive and progressive destruction of the nuclear architecture during lytic reactivation of KSHV, with significant condensation of the nuclear chromatin to the periphery of the nucleus. This was associated with extensive redistribution of the nucleolar protein UBF, which normally resides at the nucleolar FC substructure. The RNA polymerase I transcription factor UBF, the large RNA polymerase I subunit RPA194, and the vBcl-2 nucleolar partner PICT-1 [58], which also functions in ribosomal biogenesis and in attenuation of the interferon response [86,99], concomitantly redistributed and colocalized in dot-like structures. The RNA methyltransferase Fibrillarin presented a few large foci surrounded by dense chromatin, and only partially colocalized with UBF. NPM1 was found within marginal nucleolar structures or dispersed in a diffuse nucleoplasmic pattern, and Nucleolin was mostly found in RCs and at the periphery of the cell nucleus. These findings are in line with reported observations during HSV-1 infection [44]. Similarly, Nucleolin was found in the exterior of hCMV replication compartments and was suggested to be important for the proper nuclear and subnuclear localization of replication compartment protein [100,101]. Significantly, UBF did not colocalized with PML bodies and the redistribution of this protein was dependent on viral DNA replication and late gene expression.

These experiments suggest that nucleolar proteins undergo nonrandom programmed dispersion that maintains certain protein complexes, but disrupts others during productive infection. Furthermore, it is possible that the same mechanisms and functions engage the localization of UBF, RPA194 and PICT-1, which may participate in related processes during infection. It is not currently known whether these proteins acquire additional functions during lytic reactivation, or whether they continue to function according to their known roles. In addition, it is not known whether the dispersion represents a cellular response to viral infection, or whether it takes place due to the formation of replication compartments that generate condensed peripheral chromatin and thus may favor virus replication. Nevertheless, the recruitment of particular nucleolar proteins to replication compartments, such as Nucleolin, which was previously reported to participate in the formation of adenovirus and hCMV replication compartments [40,100,101], may indicate a role for these proteins in virus replication and/or assembly. Analysis of the proteome associated with adenovirus and HSV-1 genomes illustrated an enrichment of the nucleolar proteins, including components of RNA polymerase I and the processome small subunit complex, which were suggested to promote the expression of viral genes [49]. Whether lytic replication and the changes in the nucleolar architecture observed during the lytic reactivation of KSHV stimulate p53-dependent or independent stress responses, which in turn may affect cell cycle progression and host metabolic pathways, is currently unknown.

Why viral proteins localize to the nucleolus and how nucleolar perturbations affect virus infection have not been precisely explained to date. It is likely that viruses utilize the pluri-functional nature of the nucleolus to enhance virus replication or to avoid cell surveillance mechanisms, yet it is also possible that nucleolar alterations signify cellular anti-viral responses. It has also been postulated that maintaining the global production of pre-rRNA avoids activation of nucleolar stress, which can lead to cell cycle arrest and apoptosis [98]. In addition, nucleolar proteins may acquire unique functions to promote viral infection as exemplified in the case of Nucleolin, which is recruited to viral replication compartments (RCs) during HSV-1 and human cytomegalovirus (hCMV) infection [40,41,100]. Finally, nucleolar modifications might be associated with functional disturbances or alterations in rRNA transcription and processing, which could, in turn, affect protein translation.

Using different approaches, we show rRNA levels do not change during the lytic cycle. This finding is in line with previous reports in HSV-1, describing continuous association of UBF with rDNA along with colocalization with the largest RNA pol I subunit RPA43 [44], and ongoing pre-rRNA synthesis at a rate only slightly below that in uninfected cells [48]. However, unlike HSV-1 infection [48], we did not document an altered rRNA maturation pathway during the lytic cycle. Alternative modifications that may affect the turn-over of rRNAs, translation efficiency, accuracy and termination, may also alter the subsets of mRNAs targeted for translation and thus provide an additional layer of infection control.

The changes that take place on rRNA during lytic reactivation are intriguing. Alterations in ribosome composition, either by changes the ribosomal proteins or the rRNA modifications, could very well represent examples of epitranscriptomic regulation of mRNA translation [66,89,90,91,92,102]. Studies on rRNA Nm modifications in 15 human cell lines grown under different conditions demonstrated variations in the modifications of rRNA; about 30% of the Nms are invariably modified, 50% of the Nms present slightly higher variability, and only 20% of the sites show significant heterogenicity [96]. A 3D structure analysis suggests that stably modified positions are located in important functional ribosomal domains, whereas the variable positions are mostly distributed to the ribosome periphery [96]. Most Nm sites that demonstrated changes during the lytic viral cycle were not classified as variable positions in cancer cells except positions Cm2824 on LSU, Cm174 on SSU and Um14 on 5.8S rRNA. Notably, SSU-Cm174 and 5.8S-Um14 were previously described to be variable in human cell lines, including patient-derived diffuse large B-cell lymphoma (DLBCL) cell line [96,103], while Cm2824 was reported as a variable site in human cell line grown in different media and growth condition [96].

Not much is known about the effects of rRNA modifications on ribosome function. A global reduction in Nm by Fibrillarin depletion modulated the ratio between cap-dependent and IRES-mediated mRNA translation [104]. Interestingly, one of the sites that was shown to be hypo-modified upon viral reactivation is Cm174 on SSU. CRISPR-KO of SNORD45C, known to mediate this modification, affected the translation only of subsets of mRNAs [105] which were either decreased or increased. For instance, the translation of mRNAs involved in cell cycle and mitosis was down-regulated, whereas the expression of genes involved in metabolism, oxidation reduction and intracellular transport increased. It was found that the differential translation under Cm174 depletion depends on AU and GC-rich codons on the mRNAs such that those that are down-regulated harbor AU rich codons and those that are up-regulated are more GC-rich [105]. Based on this observation, we hypothesize that the de-regulated sites under lytic virus reactivation should affect translation in a similar manner, i.e., increasing or reducing the translation of proteins that support or inhibit viral propagation, respectively. Interestingly, variable levels of Nm modification were found in trypanosomes while cycling between the insect and mammalian host. However, the differentially modified Nm in trypanosomes are adjacent to functional domains such as the A, P sites and PTC [69].

Based on HydraPsiSeq, it was concluded that rRNA pseudouridylation levels differ among human cell lines and during differentiation [68]. Variable levels of modification were also observed during the cycling of trypanosomes from the insect to the mammalian host, while Ψ modifications, especially on H69, enabled the parasite to cope with the temperature shift while cycling between hosts [66]. In yeast, the depletion of several snoRNAs guiding Ψ on H69 and PTC was shown to affect the folding of the rRNA and translation [106,107]. The exact role and molecular mechanisms underlying such variability is unknown, but as with Nms, the variable Ψ levels may assist ribosome adaption to translate specific mRNAs under different conditions. The existence of variable Ψ levels is associated with pathologies and developmental processes [108]. As with Nm, rRNA pseudouridylation was shown to affect the balance between cap-dependent and IRES-mediated translation [109]. The variable Ψ and Nm modifications observed in our study are not located in the functional domains such as H69 and PTC, but certain are around functional domains. Thus, the unique changes we revealed on rRNA pattern under reactivation may preferentially drive the translation of selected viral mRNAs.

In summary, we describe major changes in the nuclear and nucleolar organization that take place during lytic reactivation of KSHV. These changes may signify cellular nucleolar stress responses, but may also occur due to the recruitment of cellular proteins to replication compartments or association with viral proteins that may have major implications on the translation of viral and host cell proteins during lytic infection. Previous studies showed that the different forms of KSHV infection are controlled through cellular and viral functions, which reprogram host epigenetic, transcriptomic, post-transcriptomic and proteomic landscapes. The ability of KSHV to affect the nucleolus and rRNA modifications constitutes a novel interaction network between viral and cellular components. The study of rRNA modifications is still in its infancy; however, the notion of altering cell fate by regulating rRNA modifications has recently begun to emerge, and its significance in viral infection is intriguing.

## Data Availability

The HydraPsiSeq and RiboMeth-seq data related to this study have been deposited in the NCBI BioProject database under the accession number PRJNA846530.

## Supplementary Materials

The following are available online at https://www.mdpi.com/article/10.3390/v14081720/s1, Figure S1: UBF, Fibrillarin and NPM1 remain in the nucleolar compartment following chemical treatment with Dox and n-Butyrate; Figure S2: Redistribution of the nucleolar proteins UBF and Fibrillarin upon lytic reactivation of KSHV in iSLK-infected cells; Figure S3: Redistribution of the nucleolar proteins UBF and Fibrillarin upon lytic reactivation of KSHV in BCBL-1; Figure S4: Changes in Ψ levels detected in KSHV-infected iSLK cells undergoing lytic reactivation; Table S1: The fold-change of Ψs in BAC16-infected iSLK/SLK-uninfected cells based on Ψ-seq for rRNA; Table S2: Complete stoichiometry of ψ sites in BAC16-infected iSLK and SLK-uninfected cells that were treated with Dox and n-Butyrate for 48 h, measured with HydraPsiSeq; Table S3: Complete stoichiometry of 2′-*O*-methylation sites in BAC16-infected iSLK and SLK-uninfected cells that were treated with Dox and n-Butyrate for 48 h, measured by RiboMeth-seq; Video S1 and S2: UBF colocalizes with viral replication compartments during lytic reactivation; Video S3: The distribution of UBF and Fibrillarin during lytic induction; Video S4: The distribution of UBF and Nucleolin during lytic induction.
