# Supplementary material for: Lytic Reactivation of the Kaposi’s Sarcoma-Associated Herpesvirus (KSHV) Is Accompanied by Major Nucleolar Alterations
Source: Viruses. 2022 Aug 4;14(8):1720. doi: 10.3390/v14081720 (PMC9412354; doi:10.3390/v14081720)
Supplement: Supplementary file 1 [file viruses-14-01720-s001.zip › Supplemental Movie Legends.pdf]

Supplemental Movie Legends

Movies S1 & S2. UBF colocalizes with viral replication compartments during lytic reactivation. 3D stacks of BAC16-infected iSLK cells that were treated with Dox and n-Butyrate for 48-hr to induce lytic reactivation. Cells were stained with anti-ORF59 and secondary Rhodamine-conjugated antibody, and subsequently with anti-UBF and anti-Rabbit Cy5-cojugated secondary antibody (Cyan). Chromatin was detected by Hoechst staining. Each Z-stack contains 31 planes at 0.5 µm steps.

Movie S3. **The distribution of UBF and Fibrillarin during lytic induction**. 3D stacks of BAC16-infected iSLK cells that were treated with Dox and n-Butyrate for 48-hr to induce lytic reactivation. Cells were stained with anti-UBF (Cyan), anti-Fibrillarin (Red) and Hoechst (Blue). Each z-stack contains 31 planes at 0.5 µm steps. The distribution of UBF indicates that lytic virus reactivation took place in both cells.

Movie S4. The distribution of UBF and Nucleolin during lytic induction. 3D stacks of BAC16-infected iSLK cells that were treated with Dox and n-Butyrate for 48-hr to induce lytic reactivation. Cells were stained with anti-UBF (Red), anti-Nucleolin (Cyan), and Hoechst (Blue). Each z-stack contains 31 planes at 0.5 µm steps. The distribution of UBF indicates that lytic virus reactivation took place in the upper cell only.
