# Supplementary material for: Lytic Reactivation of the Kaposi’s Sarcoma-Associated Herpesvirus (KSHV) Is Accompanied by Major Nucleolar Alterations
Source: Viruses. 2022 Aug 4;14(8):1720. doi: 10.3390/v14081720 (PMC9412354; doi:10.3390/v14081720)
Supplement: Supplementary file 1 [file viruses-14-01720-s001.zip › Supplementary Figures and Legends.pdf]

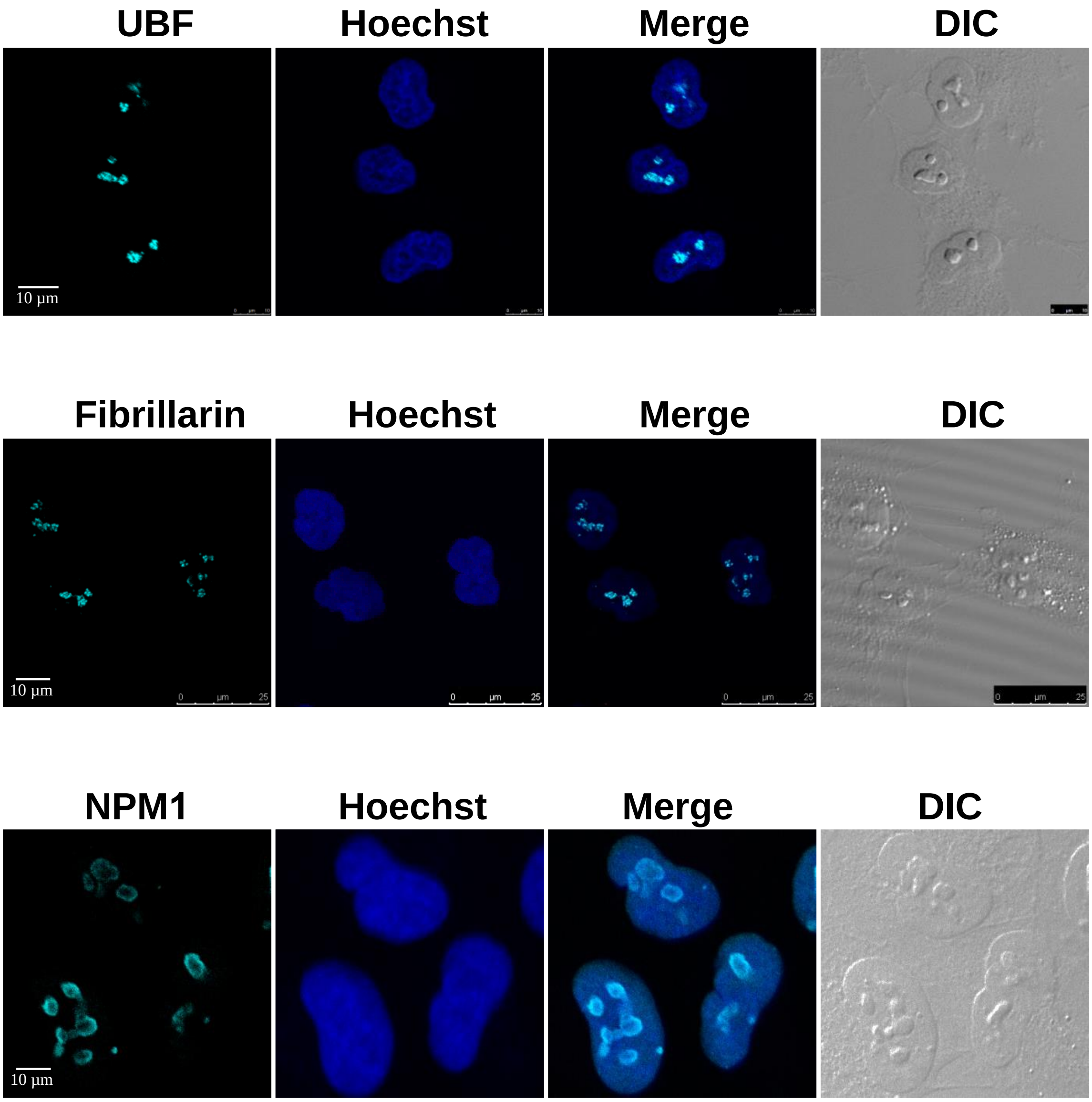

__Figure S1__. **UBF, Fibrillarin and NPM1 remain in the nucleolar compartment following chemical treatment with Dox and n-Butyrate.** To examine potential effects of the chemical treatment on the distribution of nucleolar proteins, uninfected SLK cells were treated for 48-hr with 1 µg/ml Dox and 1 mM n-Butyrate. Cells were stained with antibodies to UBF, Fibrillarin or NPM1 followed by Cy5-conjugated mouse secondary antibody for detection. The corresponding staining of nuclear DNA by Hoechst and DIC is also shown.

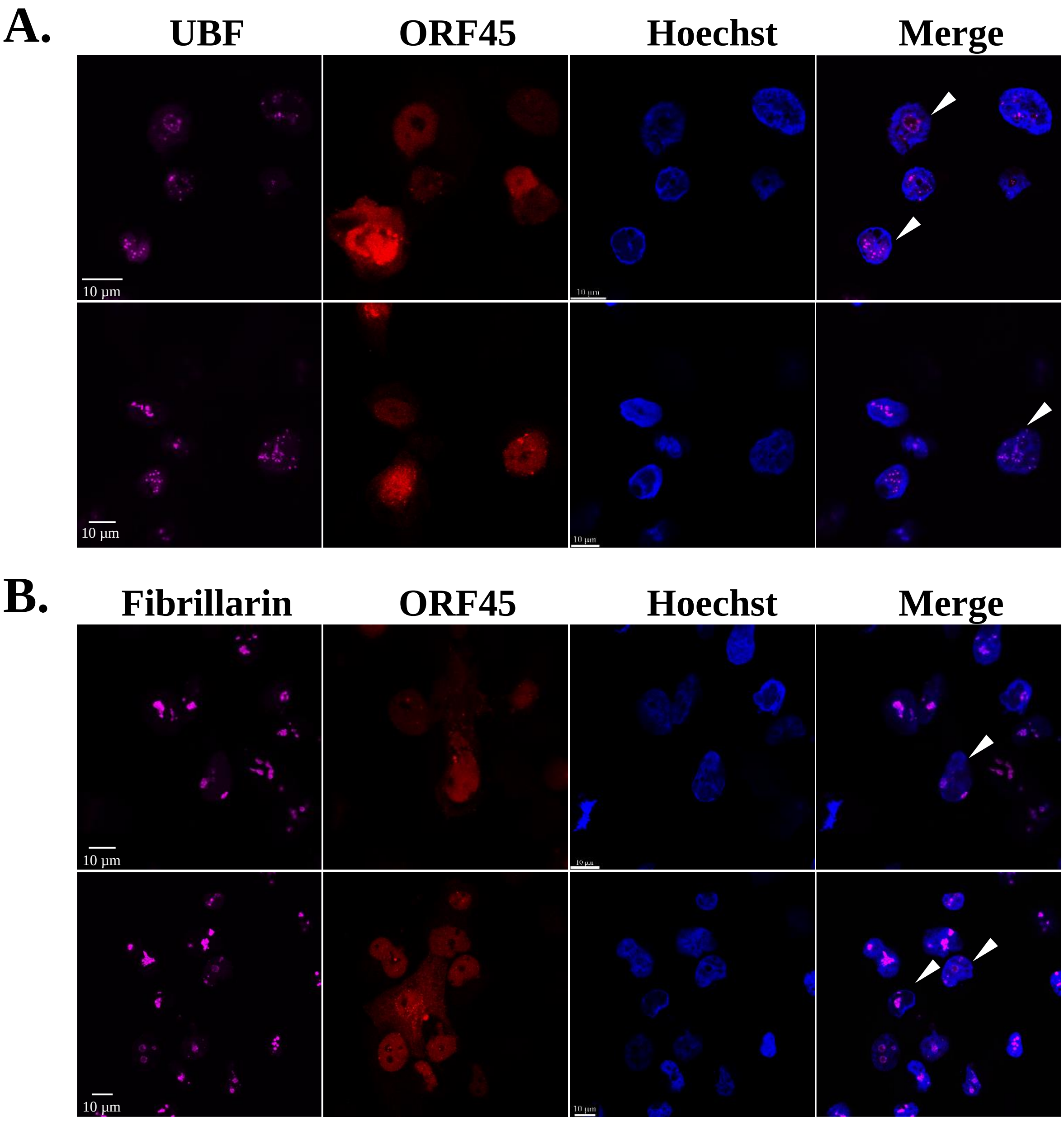

Figure S2. Redistribution of the nucleolar proteins UBF and Fibrillarin upon lytic reactivation of KSHV in iSLK-infected cells. BAC16-infected iSLK cells were treated for 48-hr with 1 µg/ml Dox and 1 mM n-Butyrate to induce viral lytic reactivation. Cells were stained with antibodies to ORF45 followed by anti-mouse Rhodamine along with UBF **(A)** or Fibrillarin **(B)** followed by anti-rabbit Alexa Fluor 647-conjugated secondary antibody. The corresponding staining of nuclear DNA by Hoechst is also shown. Arrows indicate different patterns of redistribution.

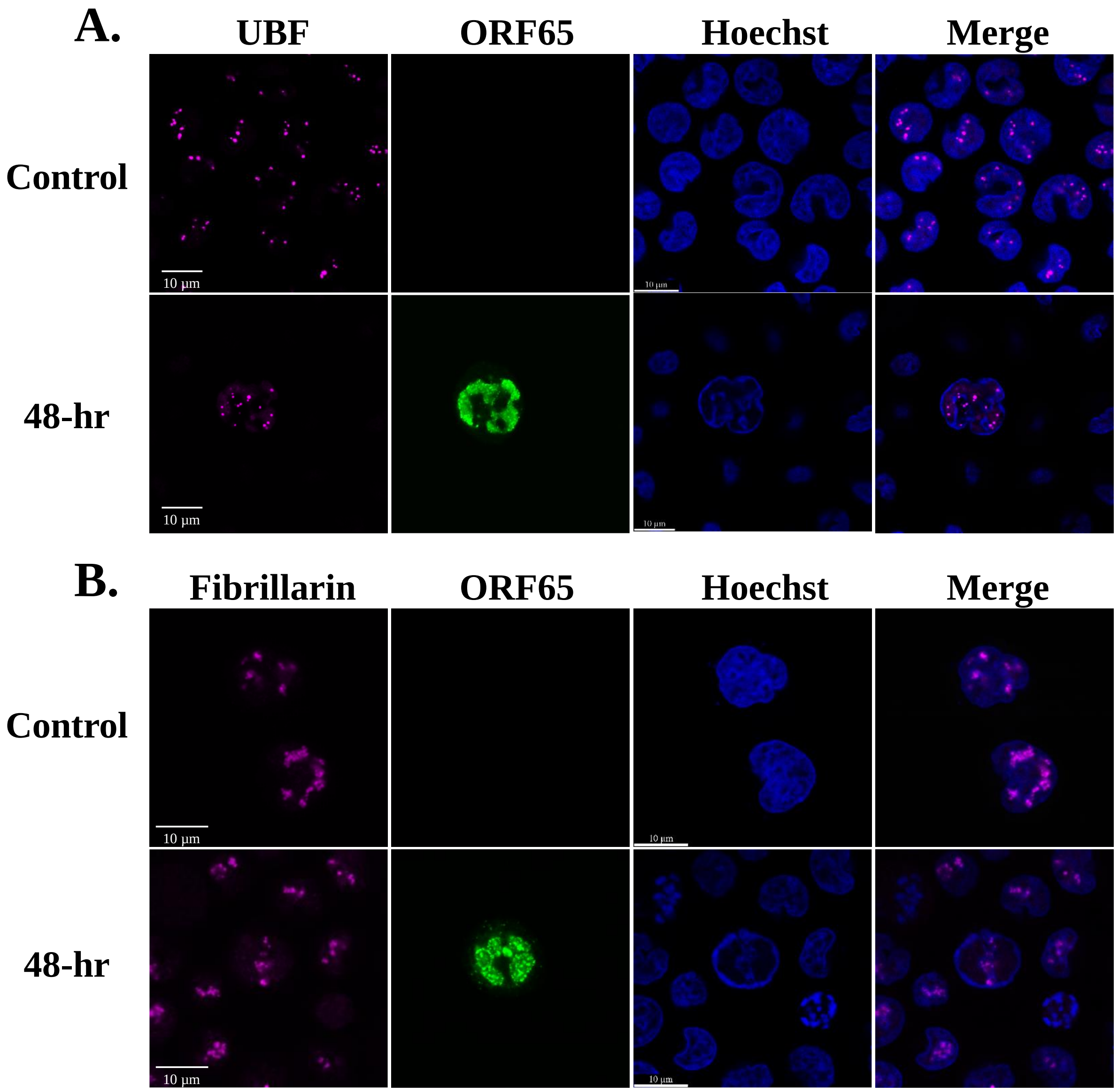

Figure S3. Redistribution of the nucleolar proteins UBF and Fibrillarin upon lytic reactivation of KSHV in BCBL-1. TREx BCBL-1 Rta cells were treated for 48-hr with 1 µg/ml Dox to induce viral lytic reactivation. Uninduced cells were used as controls. Cells were stained with antibodies to the small capsid protein ORF65 followed by anti-mouse Alexa Fluor 488-conjugated antibodies, along with UBF **(A)** or Fibrillarin **(B)** followed by anti-rabbit Alexa Fluor 647-conjugated secondary antibody. The corresponding staining of nuclear DNA by Hoechst is also shown.

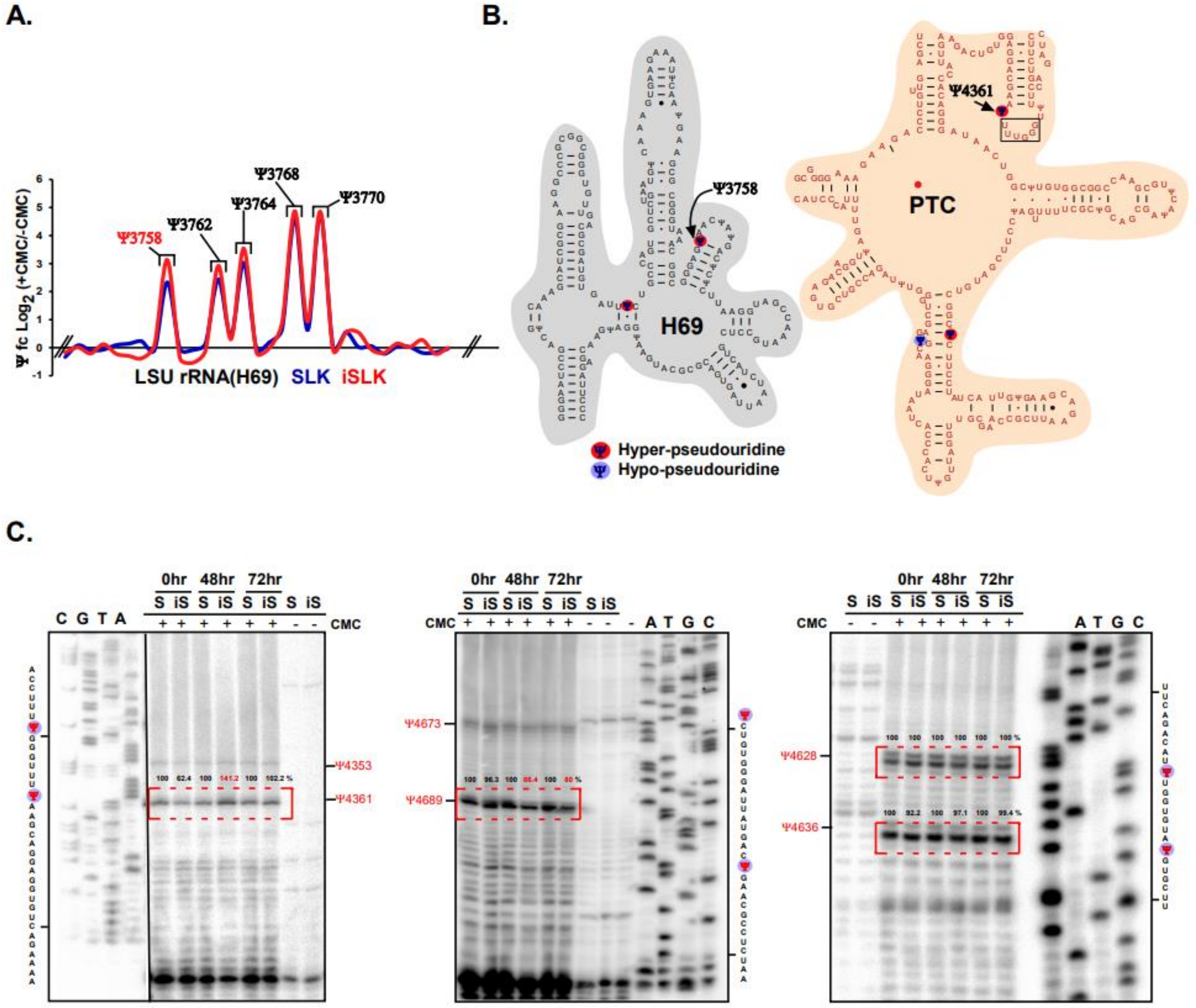

<u>Figure S4</u>. Changes in Ψ levels detected in KSHV-infected iSLK cells undergoing lytic reactivation. Uninfected SLK cells and BAC16-infected iSLK cells were treated with Dox and n-Butyrate for 48-hr. RNA-seq libraries from nuclear RNA were prepared with or without CMC (N-cyclohexyl-N9-b-(4-methylmorpholinium) ethylcarbodiimide p-tosylate). A representative line-graph of the Ψ-fc(log2) of BAC16-infected iSLK and uninfected SLK cells, representing Ψ-ratio of CMC-treated (+CMC) divided by Ψ-ratio of untreated samples (-CMC), is presented for LSU rRNA (H69 domain (A). Scheme representing the position of hyper/hypo-modified Ψ on the secondary structure of rRNA based on Ψ-seq, highlighting functional domains (H69 and PTC) of the rRNA. The structure of human rRNA was derived from (http://www.rna.ccbb.utexas.edu/) (B). Ψ verification by primer extension. Nuclear RNA, treated with CMC (+) or untreated (-), was subjected to primer extension with region-specific primers, and analyzed on 12% denaturing polyacrylamide gel. The results, along with

DNA sequencing, are presented for uninfected SLK (S) and BAC16-infected iSLK (iS) cells that were treated with Dox and n-Butyrate for the indicated time points. The positions of Ψ are indicated. As shown, increased pseudouridine level at position U4361, and decreased pseudouridine level at position U4689 was confirmed (C).
