# Supplementary material for: Lytic Reactivation of the Kaposi’s Sarcoma-Associated Herpesvirus (KSHV) Is Accompanied by Major Nucleolar Alterations
Source: Viruses. 2022 Aug 4;14(8):1720. doi: 10.3390/v14081720 (PMC9412354; doi:10.3390/v14081720)
Supplement: Supplementary file 1 [file viruses-14-01720-s001.zip › Table S1.pdf]

Table S1: The fold-change of Ψs in BAC16-infected iSLK/SLK-uninfected cells based on Ψ-seq for rRNA. Ψ-fc(log2) across four independent biological replicates for pseudouridylated sites was calculated for all Ψs. Ψ-fc(log2) >1.3 (iSLK/SLK-infected/SLK-uninfected cells that were treated with Dox and n-Butyrate for 48-hr) in at least two independent replicates was considered as hypermodified Ψ. Hypermodified and hypomodified Ψ site is highlighted in red and blue, respectively. snoRNAs predicted to guide the corresponding Ψ positions are indicated on the right column.

rRNA nt	Rep1	Rep2	Rep3	Rep4	snoRNA
SSU_U34	1.468669	0.988429	1.016245	1.0403557	SNORA76,ACA50,ACA62
SSU_U36	1.275819	0.978157	0.982736	0.9588488	SNORA69,ACA55
SSU_U93	1.099105	0.944583	1.019327	1.07740026	U23
SSU_U105	1.056816	1.138757	1.010098	1.01779137	ACA36,ACA36B,ACA50,ACA62
SSU_U109	0.958792	1.042759	0.992208	0.98786735	ACA42,ACA67
SSU_U119	0.944141	1.057933	1.017579	1.00344013	U66
SSU_U210	1.026285	0.972761	0.940047	0.91496893	ACA10
SSU_U218	0.95014	1.104273	1.01207	0.9916505	ACA31
SSU_U406	1.037276	0.932315	0.806816	0.7737492	U71A,U71B,U71C,U71D
SSU_U572	1.528347	0.908834	1.061888	1.03883899	ACA42,ACA67
SSU_U609	0.976955	0.787425	0.974681	1.02709885	ACA24
SSU_U649	0.90031	0.928624	0.991253	1.05550498	ACA46
SSU_U651	0.851527	0.92517	0.959638	0.97462104	ACA20
SSU_U681	0.988441	1.028319	1.01359	1.01117064	snoRNA unknown
SSU_U686	0.983084	1.169573	1.041192	1.01330469	ACA44
SSU_U688	0.410803	1.282688	1.162695	1.0231748	snoRNA unknown
SSU_U801	1.192798	1.879668	1.391255	1.84843555	ACA25
SSU_U814	1.301894	1.249537	0.94978	1.14805065	ACA25,ACA63
SSU_U815	1.067229	1.203814	0.99798	1.12746768	ACA28
SSU_U822	1.088918	1.131746	0.957709	0.99410917	ACA44
SSU_U863	1.00613	1.06618	0.974364	0.99931232	ACA24,ACA19
SSU_U866	1.046548	1.210732	0.963384	0.96172145	ACA28
SSU_U918	1.00129	1.018347	0.967732	0.94996017	snoRNA unknown
SSU_U966	1.330202	0.942089	1.013156	1.01581904	ACA14A,ACA14B
SSU_U1004	1.277082	0.943282	0.98685	1.04744811	ACA60,U99
SSU_U1046	1.015978	1.083074	0.932534	0.93249117	SNORA57
SSU_U1056	0.909807	1.011326	0.949366	0.92484548	ACA8
SSU_U1081	0.940439	1.079035	1.037986	0.98696823	ACA8
SSU_U1174	1.340738	1.150772	1.085431	0.9936383	ACA40

SSU_U1232	1.53262	0.979777	0.990108	0.94861696	SNORA70
SSU_U1238	1.065837	0.976392	0.975618	0.94438083	ACA5,ACA5B,ACA5C
SSU_U1244	0.862168	0.896855	0.97051	0.92693884	ACA36,ACA36B
SSU_U1248	1.242728	1.319133	0.751925	1.05759584	snoRNA unknown
SSU_U1347	0.8947	1.010253	0.99461	0.96687904	ACA4
SSU_U1367	0.93724	1.007698	0.994713	0.96560847	ACA15
SSU_U1445	0.850474	1.061705	1.03003	0.98349969	U67
SSU_U1625	1.275494	0.996099	0.937041	0.93372329	ACA5,ACA5C
SSU_U1643	1.000436	1.015723	0.961973	0.93720913	ACA41
SSU_U1692	0.98724	1.067176	0.955667	0.92945285	U70
5.8S_55	1.182568	1.039824	0.934179	0.91165603	SNORA72
5.8S_69	0.988129	0.96164	0.937108	0.88855944	SNORA69
LSU_U1536	0.966016	1.111709	0.982228	1.00161719	snoRNA unknown
LSU_U1582	1.084155	1.011051	0.996068	0.94525012	ACA7, ACA7B
LSU_U1677	1.485157	1.126585	1.057221	1.00534307	ACA56
LSU_U1683	1.309181	1.104935	1.063947	0.9922291	ACA9
LSU_U1744	1.223034	1.036014	0.906133	1.02972517	ACA52
LSU_U1779	1.292554	1.032532	0.901382	0.97947874	HBII-115
LSU_U1781	1.078263	1.030057	0.959138	0.97924746	ACA9
LSU_U1782	0.991141	1.015936	0.956222	0.95063689	ACA9
LSU_U1792	0.967819	1.013244	1.002851	0.98240009	ACA7, ACA7B
LSU_U1860	0.889636	1.019725	0.96528	0.95628518	ACA32
LSU_U1862	0.926729	1.018165	0.967578	0.94911702	snoRNA unknown
LSU_U2508	0.951663	1.037587	0.956109	0.93468343	ACA61
LSU_U3637	1.309204	0.782507	1.026932	0.97110373	ACA6
LSU_U3639	1.181594	0.899537	0.985241	0.94431825	ACA19
LSU_U3695	2.510632	1.421922	1.284121	1.330352	snoRNA unknown
LSU_U3715	5.770515	1.177458	1.141487	1.18871625	ACA27
LSU_U3730	3.576034	0.680195	0.894263	1.21109009	ACA19
LSU_U3734	1.446389	0.961407	0.908394	1.27862468	ACA31
LSU_U3758	4.965276	0.697585	0.980522	1.34410917	ACA23
LSU_U3762	3.587036	0.902314	0.875374	1.19874955	SNORA74A/U19
LSU_U3764	1.803594	0.826159	0.947037	1.16887906	SNORA74A/U19
LSU_U3768	2.695983	0.991671	0.951086	1.05243877	snoRNA unknown
LSU_U3770	1.496666	0.912813	0.936581	1.01472718	snoRNA unknown
LSU_U3818	1.390499	0.976433	0.959889	1.18033777	ACA48
LSU_U3822	0.999036	0.927757	0.96037	1.010826	ACA54
LSU_U3844	0.977596	0.941236	1.004998	1.01181004	ACA58
LSU_U3851	0.848792	0.906246	1.019531	1.00805085	E2
LSU_U3853	0.904676	1.000532	0.992033	0.97002858	E2, ACA8
LSU_U3884	0.87261	0.902521	0.868072	0.83824381	snoRNA unknown

LSU_U3920	0.93123	1.002363	0.966828	0.94316073	ACA3
LSU_U3959	0.904123	0.95046	0.999773	0.97324626	ACA3
LSU_U4293	1.121473	1.39007	1.095195	1.26083229	ACA2A, ACA2B
LSU_U4296	0.930936	1.121679	1.038018	1.18889835	snoRNA unknown
LSU_U4299	1.10639	1.181932	1.043949	1.10327164	ACA34
LSU_U4312	1.031484	1.176532	1.082566	1.17493999	ACA2A, ACA2B, ACA34
LSU_U4353	1.802119	1.250948	1.000483	1.02324973	snoRNA unknown
LSU_U4361	3.084676	1.329436	0.970958	1.02449057	ACA23, ACA64
LSU_U4403	1.648628	1.266701	0.934543	1.04975798	U65
LSU_U4420	1.161879	1.128314	0.935576	1.04606126	E3
LSU_U4423	1.033944	1.099944	0.933618	0.96002482	U68
LSU_U4431	1.317962	1.207801	0.916385	0.94074434	ACA21
LSU_U4442	1.443502	1.126367	0.948953	0.97953338	ACA16
LSU_U4457	1.444627	1.970362	0.87465	0.9030562	U65
LSU_U4471	0.983846	1.115022	0.93646	0.93606643	ACA1
LSU_U4500	1.304395	1.150632	1.194678	1.26369902	ACA21
LSU_U4521	0.648166	0.646296	0.971855	0.98418219	ACA10
LSU_U4552	0.889186	0.881244	1.02148	1.04216277	ACA27, HBI-6
LSU_U4628	0.846721	0.924661	0.83228	0.87010678	ACA17
LSU_U4636	0.758504	0.914384	0.691882	0.7558292	HBI-61
LSU_U4673	0.839703	1.000467	0.699979	0.77795188	ACA30, ACA37
LSU_U4689	0.826632	0.912667	0.681533	0.75717968	ACA17
LSU_U4972	1.209833	1.257827	1.011026	0.90332725	ACA17
LSU_U4973	1.402522	1.38188	1.060938	0.90536153	ACA43
LSU_U5001	0.838449	0.88117	1.025186	0.93655876	ACA22, ACA33
LSU_U5010	0.789932	0.900286	1.078619	0.98056112	ACA22, U64
