# Supplementary material for: Lytic Reactivation of the Kaposi’s Sarcoma-Associated Herpesvirus (KSHV) Is Accompanied by Major Nucleolar Alterations
Source: Viruses. 2022 Aug 4;14(8):1720. doi: 10.3390/v14081720 (PMC9412354; doi:10.3390/v14081720)
Supplement: Supplementary file 1 [file viruses-14-01720-s001.zip › Table S2.pdf]

<u>Table S2</u>. Complete stoichiometry of Ψ sites in BAC16-infected iSLK and SLK-uninfected cells that were treated with Dox and n-Butyrate for 48-hr, measured with HydraPsiSeq. Data indicates the fraction pseudouridylated (PsiScore) measured in three replicates. Data are presented as mean +/- S.E.R. Highlighted in red are FC > 1.2 and highlighted in green are FC<0.8.

rRNA	Ψ site	Ψ -seq	HydraPsiSeq	Infected	Uninfected	Inf/Uninf
SSU	Ψ34			0.92+/-0.00	0.92+/-0.01	1.00
SSU	Ψ36			0.92+/-0.01	0.93+/-0.01	1.00
SSU	Ψ93			0.81+/-0.03	0.88+/-0.02	0.92
SSU	Ψ105			0.82+/-0.04	0.83+/-0.02	0.99
SSU	Ψ109			0.90+/-0.02	0.89+/-0.01	1.01
SSU	Ψ119			0.90+/-0.01	0.91+/-0.01	0.99
SSU	Ψ210			0.71+/-0.02	0.86+/-0.02	0.83
SSU	Ψ218			0.78+/-0.01	0.79+/-0.00	0.99
SSU	Ψ406			0.48+/-0.01	0.79+/-0.01	0.61
SSU	Ψ572			0.80+/-0.01	0.81+/-0.02	0.99
SSU	Ψ609			0.91+/-0.01	0.91+/-0.01	1.01
SSU	Ψ649			0.65+/-0.01	0.69+/-0.01	0.93
SSU	Ψ651			0.76+/-0.02	0.76+/-0.01	1.00
SSU	Ψ681			0.71+/-0.01	0.72+/-0.00	1.00
SSU	Ψ686			0.93+/-0.00	0.93+/-0.00	1.00
SSU	Ψ801			0.97+/-0.00	0.96+/-0.01	1.00
SSU	Ψ814			0.78+/-0.01	0.75+/-0.01	1.04
SSU	Ψ815			0.92+/-0.01	0.91+/-0.01	1.01
SSU	Ψ822			0.65+/-0.02	0.68+/-0.01	0.96
SSU	Ψ863			0.94+/-0.01	0.95+/-0.00	0.99
SSU	Ψ866			0.73+/-0.01	0.75+/-0.04	0.97
SSU	Ψ966 Ψ			0.96+/-0.00	0.96+/-0.00	1.00
SSU	Ψ1004			0.82+/-0.00	0.81+/-0.01	1.01
SSU	Ψ1056			0.93+/-0.01	0.93+/-0.01	0.99
SSU	Ψ1081			0.92+/-0.01	0.92+/-0.01	1.00
SSU	Ψ1174			0.91+/-0.00	0.91+/-0.00	1.01
SSU	Ψ1238			0.46+/-0.02	0.39+/-0.09	1.19
SSU	Ψ1244			0.98+/-0.00	0.99+/-0.00	1.00
SSU	Ψ1347			0.76+/-0.04	0.71+/-0.02	1.07
SSU	Ψ1367			0.74+/-0.02	0.73+/-0.01	1.01
SSU	Ψ1445			0.87+/-0.00	0.87+/-0.01	1.01
SSU	Ψ1625			0.90+/-0.00	0.90+/-0.01	1.00
SSU	Ψ1643			0.92+/-0.01	0.94+/-0.00	0.97
SSU	Ψ1692			0.87+/-0.01	0.86+/-0.00	1.00
5.8S	Ψ55			0.82+/-0.01	0.89+/-0.00	0.92
5.8S	Ψ69			0.93+/-0.00	0.93+/-0.00	1.00
LSU	Ψ1536			0.90+/-0.01	0.90+/-0.01	1.00

LSU	Ψ1582			0.95+/-0.00	0.97+/-0.01	0.99
LSU	Ψ1677			0.96+/-0.00	0.97+/-0.00	0.99
LSU	Ψ1683			0.94+/-0.00	0.94+/-0.01	0.99
LSU	Ψ1744			0.85+/-0.01	0.85+/-0.01	1.01
LSU	Ψ1782			0.82+/-0.01	0.78+/-0.03	1.06
LSU	Ψ1792			0.90+/-0.00	0.90+/-0.00	1.00
LSU	Ψ1860			0.89+/-0.01	0.88+/-0.01	1.01
LSU	Ψ1862			0.70+/-0.01	0.70+/-0.01	0.99
LSU	Ψ2508			0.86+/-0.02	0.90+/-0.00	0.96
LSU	Ψ3637			0.94+/-0.00	0.95+/-0.00	0.99
LSU	Ψ3639			0.70+/-0.02	0.72+/-0.04	0.98
LSU	Ψ3695	Hyper		0.80+/-0.01	0.82+/-0.01	0.98
LSU	Ψ3715			0.95+/-0.00	0.95+/-0.00	1.00
LSU	Ψ3758	Hyper		0.92+/-0.01	0.92+/-0.01	1.01
LSU	Ψ3770			0.72+/-0.01	0.79+/-0.04	0.91
LSU	Ψ3844			0.94+/-0.00	0.94+/-0.00	1.00
LSU	Ψ3851			0.85+/-0.01	0.87+/-0.01	0.98
LSU	Ψ3853			0.91+/-0.00	0.91+/-0.01	1.00
LSU	Ψ3884		Hypo	0.27+/-0.03	0.50+/-0.05	0.54
LSU	Ψ3920			0.81+/-0.01	0.80+/-0.01	1.01
LSU	Ψ3959			0.95+/-0.00	0.95+/-0.00	1.00
LSU	Ψ4299			0.71+/-0.01	0.70+/-0.01	1.02
LSU	Ψ4312			0.92+/-0.01	0.93+/-0.00	1.00
LSU	Ψ4353			0.88+/-0.00	0.87+/-0.00	1.01
LSU	Ψ4361	hyper		0.71+/-0.02	0.75+/-0.01	0.94
LSU	Ψ4431			0.92+/-0.02	0.93+/-0.02	0.99
LSU	Ψ4442			0.91+/-0.00	0.91+/-0.00	1.00
LSU	Ψ4457	hyper		0.95+/-0.01	0.95+/-0.00	1.00
LSU	Ψ4471			0.92+/-0.00	0.89+/-0.02	1.04
LSU	Ψ4500			0.78+/-0.07	0.76+/-0.04	1.03
LSU	Ψ4521	hypo		0.98+/-0.02	1.00+/-0.00	0.98
LSU	Ψ4532			0.94+/-0.00	0.94+/-0.02	1.00
LSU	Ψ4552			0.94+/-0.01	0.94+/-0.01	1.00
LSU	Ψ4576			0.80+/-0.01	0.79+/-0.03	1.01
LSU	Ψ4579			0.75+/-0.01	0.78+/-0.01	0.96
LSU	Ψ4628			0.74+/-0.02	0.72+/-0.01	1.02
LSU	Ψ4636	hypo	Hyper	0.24+/-0.04	0.18+/-0.08	1.35
LSU	Ψ4673			0.81+/-0.01	0.78+/-0.01	1.04
LSU	Ψ4689	hypo		0.82+/-0.02	0.83+/-0.01	0.99
LSU	Ψ4972			0.86+/-0.00	0.85+/-0.01	1.01
LSU	Ψ4973	Hyper	Hyper	0.31+/-0.02	0.25+/-0.01	1.25
LSU	Ψ5001			0.77+/-0.01	0.77+/-0.01	1.00
LSU	Ψ5010			0.86+/-0.01	0.89+/-0.01	0.97
