# Supplementary material for: Lytic Reactivation of the Kaposi’s Sarcoma-Associated Herpesvirus (KSHV) Is Accompanied by Major Nucleolar Alterations
Source: Viruses. 2022 Aug 4;14(8):1720. doi: 10.3390/v14081720 (PMC9412354; doi:10.3390/v14081720)
Supplement: Supplementary file 1 [file viruses-14-01720-s001.zip › Table S3.pdf]

Table S3. Complete stoichiometry of 2'-*O*-methylation sites in BAC16-infected iSLK and SLK-uninfected cells that were treated with Dox and n-Butyrate for 48-hr, measured by RiboMeth-seq. Data indicates the fraction methylated (Score-C) measured in three replicates. Data are presented as mean +/- S.E.R. The identity of Nm and the snoRNA assigned is indicated. Highlighted in red are FC > 1.2.

Nm	snoRNA	Uninfected	Infected	Infected/Uninfected	Uninfed/Infected
SSU_Am27	snoRD27	0.85+/-0.01	0.85+/-0.01	1.00	1.00
SSU_Am99	snoRD57	0.92+/-0.00	0.90+/-0.00	0.98	1.02
SSU_Um116	snoRD42	0.74+/-0.01	0.73+/-0.01	0.99	1.01
SSU_Um121	snoRD4A/B	0.78+/-0.01	0.77+/-0.00	0.99	1.01
SSU_Am159	snoRD45A/C	0.88+/-0.00	0.85+/-0.02	0.96	1.04
SSU_Am166	snoRD44	0.82+/-0.00	0.79+/-0.00	0.97	1.03
SSU_Um172	snoRD45A/B	0.68+/-0.01	0.64+/-0.03	0.93	1.08
SSU_Cm174	snoRD45C	0.41+/-0.08	0.33+/-0.07	0.79	1.26
SSU_Um354	snoRD90	0.33+/-0.01	0.28+/-0.03	0.84	1.18
SSU_Um428	snoRD68	0.74+/-0.04	0.74+/-0.03	1.00	1.00
SSU_Gm436	snoRD100	0.63+/-0.01	0.58+/-0.03	0.92	1.09
SSU_Cm462	snoRD14A/B/CDE	0.54+/-0.03	0.58+/-0.03	1.06	0.95
SSU_Am468	snoRD83A	0.84+/-0.01	0.83+/-0.00	0.99	1.01
SSU_Am484	snoRD16	0.94+/-0.00	0.94+/-0.01	1.00	1.00
SSU_Gm509	snoRD11/B	0.88+/-0.01	0.86+/-0.01	0.98	1.02
SSU_Am512	snoRD70	0.77+/-0.02	0.75+/-0.03	0.98	1.02
SSU_Cm517	snoRD56/B	0.89+/-0.01	0.88+/-0.01	0.99	1.01
SSU_Am576	snoRD93	0.82+/-0.01	0.83+/-0.01	1.01	0.99
SSU_Am590	snoRD62A/B	0.53+/-0.02	0.55+/-0.03	1.04	0.97
SSU_Um627	snoRD65	0.44+/-0.01	0.40+/-0.06	0.90	1.11
SSU_Gm644	snoRD54	0.82+/-0.01	0.82+/-0.02	1.00	1.00
SSU_Am668	snoRD36A/B	0.81+/-0.01	0.83+/-0.02	1.03	0.97
SSU_Gm683	snoRD19/B	0.79+/-0.01	0.77+/-0.02	0.97	1.03
SSU_Cm797	snoRDZL107?	0.31+/-0.02	0.29+/-0.01	0.92	1.09
SSU_Um799	snoRD105/B	0.39+/-0.03	0.41+/-0.10	1.05	0.95
SSU_Gm867	snoRD98	0.66+/-0.01	0.61+/-0.01	0.92	1.09
SSU_Am1031	snoRD59A/B	0.92+/-0.01	0.92+/-0.00	1.01	0.99
SSU_Cm1272	snoRD66	0.72+/-0.00	0.71+/-0.02	0.98	1.02
SSU_Um1288	snoRD110	0.89+/-0.01	0.88+/-0.01	0.99	1.01
SSU_Um1326	snoRD33	0.59+/-0.03	0.62+/-0.02	1.04	0.96
SSU_Am1383	snoRD30?	0.89+/-0.00	0.89+/-0.00	1.00	1.00
SSU_Cm1391	snoRD28	0.80+/-0.01	0.80+/-0.02	1.00	1.00
SSU_Um1442	snoRD61	0.74+/-0.03	0.70+/-0.02	0.94	1.06
SSU_Gm1447	snoRD127	0.48+/-0.02	0.45+/-0.04	0.94	1.07
SSU_Gm1490	snoRD25	0.85+/-0.00	0.84+/-0.01	0.99	1.01
SSU_Am1678	snoRD82	0.62+/-0.04	0.65+/-0.03	1.05	0.95
SSU_Cm1703	snoRD43	0.43+/-0.01	0.42+/-0.03	0.99	1.01
SSU_Um1804	snoRD20	0.87+/-0.01	0.85+/-0.03	0.98	1.02

5.8S_Um14	snoRD71	0.32+/-0.04	0.23+/-0.05	0.71	1.42
5.8S_Gm75	snoRD96A/B	0.42+/-0.04	0.46+/-0.03	1.10	0.91
LSU_Am398	snoRD26	0.32+/-0.05	0.38+/-0.04	1.20	0.83
LSU_Am400	snoRD81	0.68+/-0.02	0.69+/-0.02	1.01	0.99
LSU_Gm1316	snoRD21	0.45+/-0.03	0.40+/-0.03	0.88	1.13
LSU_Am1323	snoRD126?	0.70+/-0.02	0.68+/-0.03	0.97	1.03
LSU_Am1326	snoRD18A/B/C	0.90+/-0.00	0.90+/-0.00	1.00	1.00
LSU_Cm1340	snoRD104	0.55+/-0.01	0.56+/-0.02	1.02	0.98
LSU_Gm1522	snoRD2	0.90+/-0.01	0.90+/-0.00	1.00	1.00
LSU_Am1524	snoRD32A/B/snoRD51	0.55+/-0.05	0.53+/-0.06	0.97	1.03
LSU_Am1534	snoRD77/snoRD80	0.85+/-0.01	0.85+/-0.01	1.00	1.00
LSU_Gm1625	snoRD80	0.60+/-0.04	0.60+/-0.05	1.01	0.99
LSU_Gm1760	snoRD73A	0.52+/-0.04	0.51+/-0.05	0.97	1.03
LSU_Am1871	snoRD38A/B	0.95+/-0.00	0.95+/-0.00	0.99	1.01
LSU_Cm1881	snoRD48?	0.49+/-0.03	0.48+/-0.03	0.97	1.04
LSU_Cm2351	snoRD24	0.61+/-0.01	0.61+/-0.01	0.99	1.01
LSU_Am2363	snoRD76	0.69+/-0.01	0.68+/-0.03	0.99	1.01
LSU_Gm2364	snoRD_Unknown	0.50+/-0.02	0.49+/-0.07	0.98	1.02
LSU_Am2401	snoRD68	0.68+/-0.01	0.63+/-0.02	0.92	1.09
LSU_Um2415	snoRD143/144?	0.57+/-0.02	0.57+/-0.03	1.00	1.00
LSU_Cm2422	snoRD5	0.54+/-0.02	0.56+/-0.02	1.03	0.97
LSU_Gm2424	snoRD6	0.92+/-0.00	0.91+/-0.00	0.99	1.01
LSU_Am2787	snoRD99	0.69+/-0.01	0.66+/-0.02	0.96	1.05
LSU_Cm2804	snoRD55	0.71+/-0.01	0.66+/-0.02	0.94	1.07
LSU_Am2815	snoRD95	0.79+/-0.01	0.81+/-0.01	1.02	0.98
LSU_Cm2824	snoRD95	0.24+/-0.08	0.34+/-0.09	1.42	0.71
LSU_Um2837	snoRD34	0.72+/-0.02	0.75+/-0.03	1.04	0.96
LSU_Cm2861	snoRD50A/B	0.84+/-0.02	0.83+/-0.02	0.99	1.01
LSU_Gm2876	snoRD50A/B	0.72+/-0.03	0.69+/-0.03	0.97	1.04
LSU_Cm3701	snoRD88A/B/C	0.84+/-0.00	0.84+/-0.01	1.00	1.00
LSU_Am3718	snoRD37	0.87+/-0.01	0.85+/-0.01	0.97	1.03
LSU_Am3724	snoRD36C	0.91+/-0.00	0.90+/-0.01	0.99	1.01
LSU_Gm3744	snoRD87	0.63+/-0.02	0.60+/-0.01	0.94	1.06
LSU_Am3760	snoRD46	0.77+/-0.01	0.79+/-0.00	1.03	0.97
LSU_Am3785	snoRD15A/B	0.71+/-0.01	0.70+/-0.01	0.98	1.02
LSU_Gm3792	snoRD15A/B	0.85+/-0.00	0.85+/-0.00	0.99	1.01
LSU_Cm3808	snoRD10	0.82+/-0.02	0.81+/-0.01	0.99	1.01
LSU_Um3818	snoRD17	0.64+/-0.02	0.65+/-0.00	1.01	0.99
LSU_Am3825	snoRD30	0.81+/-0.01	0.82+/-0.01	1.01	0.99
LSU_Am3830	snoRD79	0.71+/-0.01	0.71+/-0.01	1.00	1.00
LSU_Cm3841	snoRD74	0.38+/-0.01	0.39+/-0.01	1.03	0.97
LSU_Am3867	snoRD92	0.71+/-0.01	0.68+/-0.00	0.95	1.05
LSU_Cm3887	snoRD47	0.63+/-0.02	0.65+/-0.04	1.03	0.97
LSU_Gm3899	snoRD12/B/SNORA45	0.58+/-0.02	0.60+/-0.01	1.03	0.97
LSU_Um3925	snoRD52	0.70+/-0.01	0.69+/-0.02	0.99	1.01
LSU_Gm3944	snoRD111/B	0.85+/-0.01	0.84+/-0.01	0.99	1.01

LSU_Gm4042	snoRD102	0.79+/-0.02	0.76+/-0.02	0.96	1.04
LSU_Cm4054	snoRD75	0.20+/-0.00	0.24+/-0.03	1.16	0.86
LSU_Um4227	snoRD_Unknown	0.78+/-0.01	0.78+/-0.02	1.00	1.00
LSU_Gm4228	snoRD58A/B/C	0.70+/-0.02	0.71+/-0.03	1.01	0.99
LSU_Um4306	snoRD41	0.54+/-0.03	0.50+/-0.01	0.92	1.08
LSU_Gm4370	snoRD60	0.79+/-0.02	0.80+/-0.01	1.01	0.99
LSU_Gm4392	snoRD1A/B/C	0.31+/-0.03	0.34+/-0.01	1.11	0.90
LSU_Cm4456	snoRD49A/B	0.57+/-0.03	0.60+/-0.01	1.06	0.94
LSU_Gm4494	snoRD69	0.93+/-0.01	0.92+/-0.01	0.98	1.02
LSU_Um4498	snoRD62AB	0.90+/-0.01	0.89+/-0.02	0.99	1.01
LSU_Gm4499	snoRD75/hSbp1	0.87+/-0.01	0.87+/-0.02	1.01	0.99
LSU_Am4523	snoRD29	0.93+/-0.02	0.94+/-0.02	1.01	0.99
LSU_Cm4536	snoRD35A/B	0.91+/-0.00	0.91+/-0.00	1.00	1.00
LSU_Am4571	snoRD63	0.49+/-0.03	0.53+/-0.02	1.08	0.92
LSU_Am4590	snoRD119	0.21+/-0.03	0.27+/-0.03	1.29	0.78
LSU_Um4620	snoRD72	0.78+/-0.02	0.79+/-0.02	1.01	0.99
LSU_Gm4623	snoRD78	0.60+/-0.04	0.65+/-0.01	1.08	0.93
LSU_Gm4637	snoRD121A/B	0.49+/-0.03	0.52+/-0.00	1.07	0.93
