# Supplementary material for: Lytic Reactivation of the Kaposi’s Sarcoma-Associated Herpesvirus (KSHV) Is Accompanied by Major Nucleolar Alterations
Source: Viruses. 2022 Aug 4;14(8):1720. doi: 10.3390/v14081720 (PMC9412354; doi:10.3390/v14081720)
Supplement: Supplementary file 1 [file viruses-14-01720-s001.zip › viruses-1794797-Figures-supplementary.pdf]

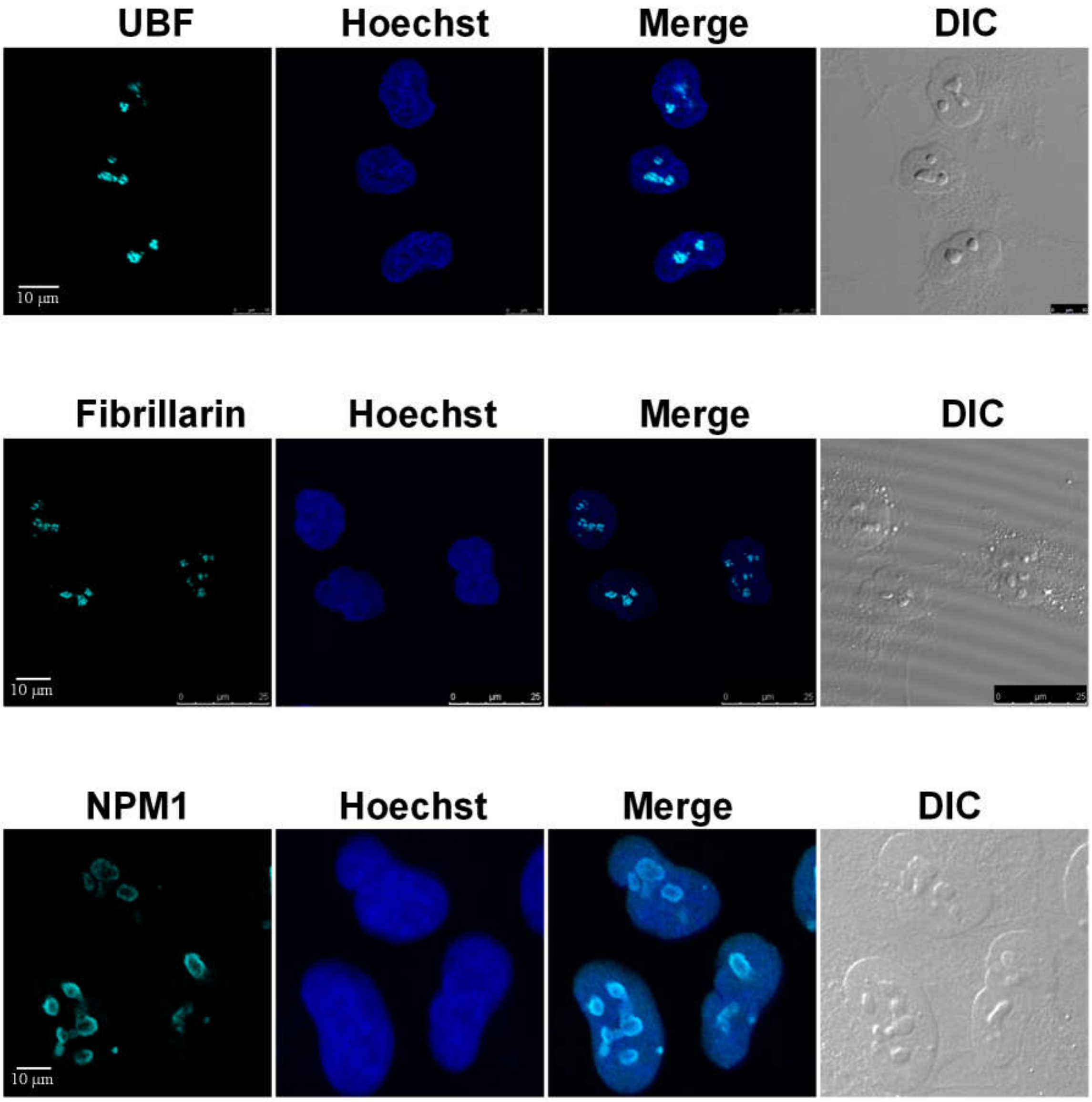

<u>Figure S1</u>. **UBF, Fibrillarin and NPM1 remain in the nucleolar compartment following chemical treatment with Dox and n-Butyrate.** To examine potential effects of the chemical treatment on the distribution of nucleolar proteins, uninfected SLK cells were treated for 48-hr with 1 μg/ml Dox and 1 mM n-Butyrate. Cells were stained with antibodies to UBF, Fibrillarin or NPM1 followed by Cy5-conjugated mouse secondary antibody for detection. The corresponding staining of nuclear DNA by Hoechst and DIC is also shown.

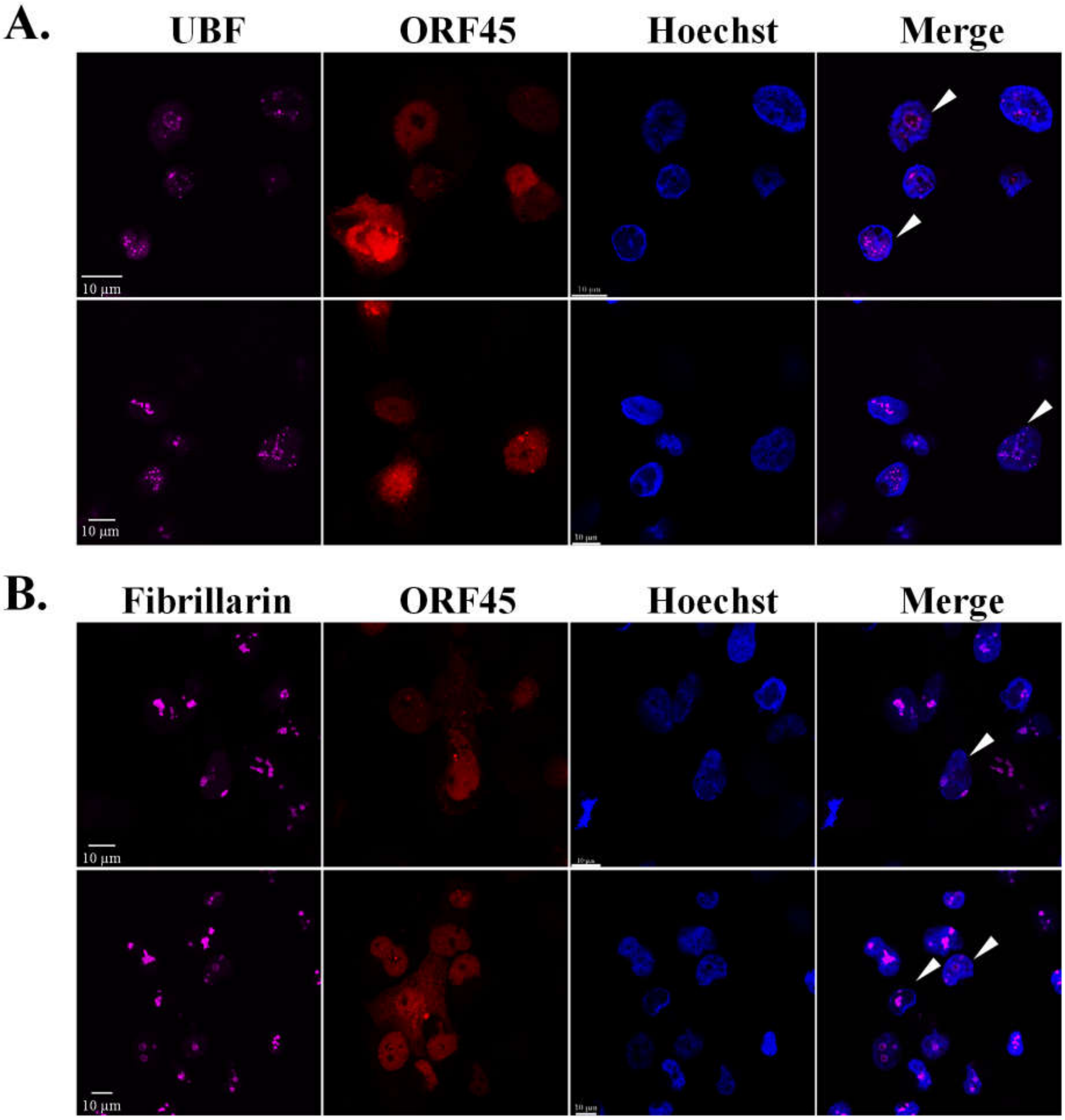

<u>Figure S2</u>. Redistribution of the nucleolar proteins UBF and Fibrillarin upon lytic reactivation of KSHV in iSLK-infected cells. BAC16-infected iSLK cells were treated for 48-hr with 1 μg/ml Dox and 1 mM n-Butyrate to induce viral lytic reactivation. Cells were stained with antibodies to ORF45 followed by anti-mouse Rhodamine along with UBF **(A)** or Fibrillarin **(B)** followed by anti-rabbit Alexa Fluor 647-conjugated secondary antibody. The corresponding staining of nuclear DNA by Hoechst is also shown. Arrows indicate different patterns of redistribution.

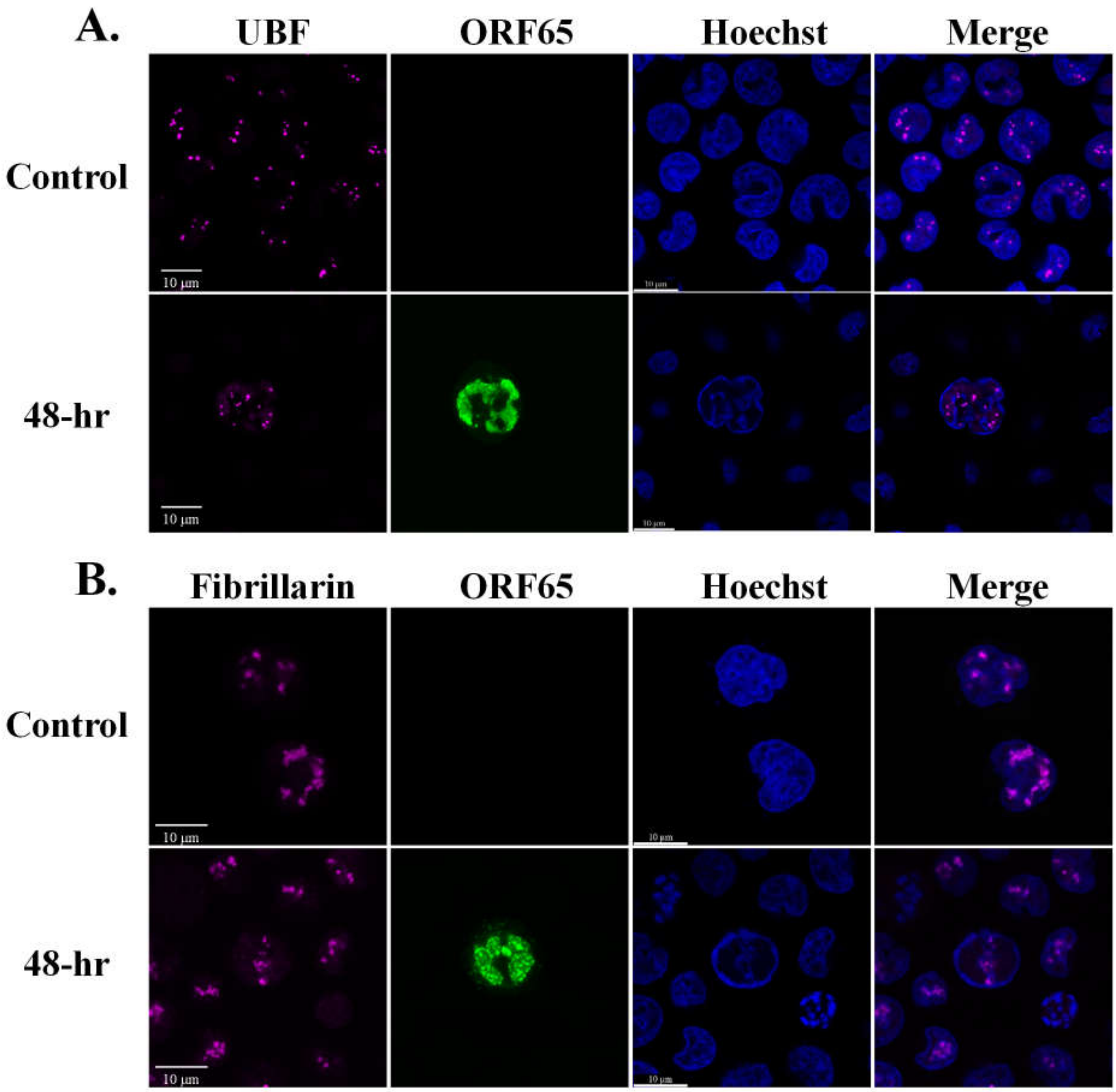

<u>Figure S3</u>. **Redistribution of the nucleolar proteins UBF and Fibrillarin upon lytic reactivation of KSHV in BCBL-1.** TREx BCBL-1 Rta cells were treated for 48-hr with 1 µg/ml Dox to induce viral lytic reactivation. Uninduced cells were used as controls. Cells were stained with antibodies to the small capsid protein ORF65 followed by anti-mouse Alexa Fluor 488-conjugated antibodies, along with UBF **(A)** or Fibrillarin **(B)** followed by anti-rabbit Alexa Fluor 647-conjugated secondary antibody. The corresponding staining of nuclear DNA by Hoechst is also shown.

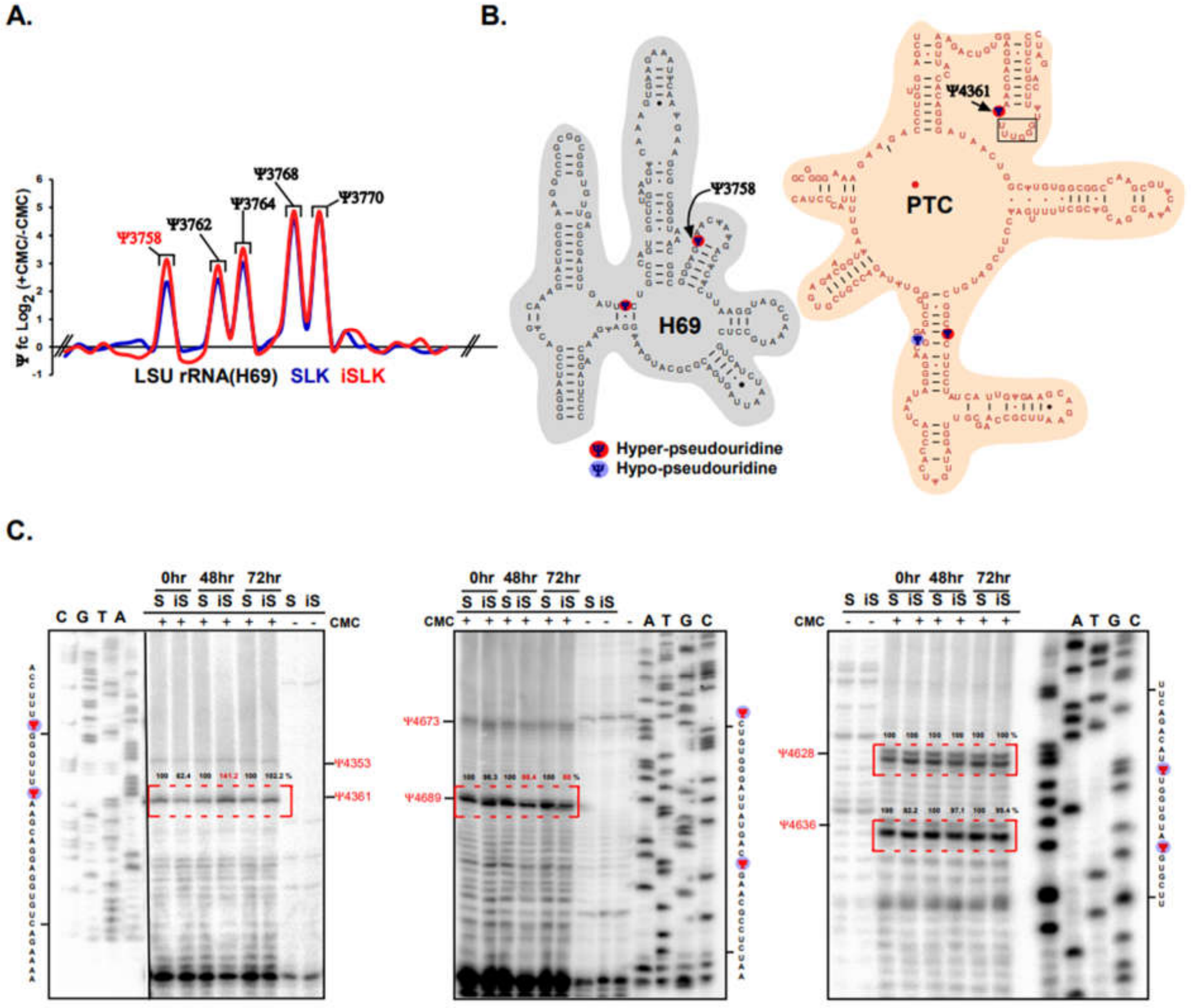

<u>Figure S4</u>. Changes in Ψ levels detected in KSHV-infected iSLK cells undergoing lytic reactivation. Uninfected SLK cells and BAC16-infected iSLK cells were treated with Dox and n-Butyrate for 48-hr. RNA-seq libraries from nuclear RNA were prepared with or without CMC (N-cyclohexyl-N9-b-(4-methylmorpholinium) ethylcarbodiimide p-tosylate). A representative line-graph of the Ψ-fc(log2) of BAC16-infected iSLK and uninfected SLK cells, representing Ψ-ratio of CMC-treated (+CMC) divided by Ψ-ratio of untreated samples (-CMC), is presented for LSU rRNA (H69 domain (A). Scheme representing the position of hyper/hypo-modified Ψ on the secondary structure of rRNA based on Ψ-seq, highlighting functional domains (H69 and PTC) of the rRNA. The structure of human rRNA was derived from (http://www.rna.ccbb.utexas.edu/) (B). Ψ verification by primer extension. Nuclear RNA, treated with CMC (+) or untreated (-), was subjected to primer extension with

region-specific primers, and analyzed on 12% denaturing polyacrylamide gel. The results, along with DNA sequencing, are presented for uninfected SLK (S) and BAC16-infected iSLK (iS) cells that were treated with Dox and n-Butyrate for the indicated time points. The positions of Ψ are indicated. As shown, increased pseudouridine level at position U4361, and decreased pseudouridine level at position U4689 was confirmed (C).
